# Adipose-derived mesenchymal stem cell-exosomes attenuate lipopolysaccharide-induced acute lung injury in mice by regulating the TGFBR2/Smad4 axis to suppress NLRP3-mediated macrophage M1 polarization and pyroptosis

**DOI:** 10.3389/fimmu.2026.1739115

**Published:** 2026-09-14

**Authors:** Jie Li, Huan Kang, Baolong Wang, Fangqiang Zou, Wei Liu, Weixin Guo, Xinliang Liao

**Affiliations:** 1 Department of Geriatric Intensive Care Medicine, Guangdong Provincial Geriatrics Institute, Guangdong Provincial People’s Hospital, Guangdong Academy of Medical Sciences, Southern Medical University, Guangzhou, Guangdong, China; 2 Department of Intensive Care Medicine, Geriatric Center, National Regional Medical Center, Guangdong Provincial People’s Hospital Ganzhou Hospital, Ganzhou Municipal Hospital, Ganzhou, Jiangxi, China; 3 Department of Geriatrics, Geriatric Center, National Regional Medical Center, Guangdong Provincial People’s Hospital Ganzhou Hospital, Ganzhou Municipal Hospital, Ganzhou, Jiangxi, China

**Keywords:** adipose-derived mesenchymal stem cells, exosomes, LPS-induced acute lung injury, lung epithelial cells, macrophages, microRNA-19b-3p, pyroptosis, transforming growth factor beta receptor II

## Abstract

**Objective:**

This study investigated whether adipose-derived mesenchymal stem cells-exosomes (ADMSC-Exo) alleviate lipopolysaccharide (LPS)-induced acute lung injury (ALI) by regulating NLRP3 inflammasome-mediated macrophage M1 polarization and pyroptosis through the miR-19b-3p/TGFBR2/Smad4 signaling axis.

**Methods:**

An LPS-induced mouse model of ALI was established and treated with ADMSC-Exo or adenoviral TGFBR2 overexpression (Ad-TGFBR2). Lung histopathology, lung wet-to-dry (W/D) weight ratio, macrophage polarization, and pyroptosis were evaluated. *In vitro*, macrophages with TGFBR2 knockdown or overexpression were stimulated with LPS and treated with ADMSC-Exo. Lung epithelial cells were subsequently cultured with macrophage-conditioned medium. The expression of miR-19b-3p, TGFBR2, mothers against decapentaplegic homolog 4 (Smad4), and NOD-like receptor protein 3 (NLRP3), together with macrophage polarization, pyroptosis, and epithelial cell apoptosis, was assessed. Predicted molecular interactions were validated using bioinformatic analyses and the JASPAR database.

**Results:**

ADMSC-Exo alleviated LPS-induced lung injury by preserving alveolar architecture and reducing lung injury scores and the W/D ratio. They inhibited M1 macrophage polarization and pyroptosis both *in vivo* and *in vitro*, thereby decreasing apoptosis of lung epithelial cells. Mechanistically, ADMSC-Exo-delivered miR-19b-3p, which targeted TGFBR2 and inhibited TGFBR2/Smad4 signaling, resulting in transcriptional suppression of NLRP3 expression. Inhibition of miR-19b-3p or overexpression of Smad4/NLRP3 partially abolished the protective effects of ADMSC-Exo. Collectively, ADMSC-Exo attenuated LPS-induced ALI by suppressing NLRP3-mediated macrophage M1 polarization and pyroptosis through the miR-19b-3p/TGFBR2/Smad4 signaling axis.

**Conclusion:**

ADMSC-derived exosomes alleviate LPS-induced acute lung injury by delivering miR-19b-3p to inhibit the TGFBR2/Smad4 signaling pathway, thereby suppressing NLRP3-mediated macrophage M1 polarization and pyroptosis and reducing lung epithelial cell apoptosis.

## 1 Introduction

Sepsis is a life-threatening syndrome characterized by organ dysfunction resulting from a dysregulated host response to infection (1, 2). Among the organs affected, the lung is particularly susceptible during the early stage of sepsis (3), frequently progressing to acute lung injury (ALI) or acute respiratory distress syndrome (ARDS). Increasing evidence indicates that dysregulated forms of programmed cell death, including autophagy, ferroptosis, and pyroptosis, contribute to the pathogenesis of lipopolysaccharide (LPS)-induced ALI, and therapeutic strategies targeting these pathways have shown protective effects in experimental models of LPS or cecal ligation and puncture-induced lung injury (4). Notably, macrophages play a central role in the initiation and progression of ALI/ARDS by orchestrating inflammatory responses (5). Excessive M1 macrophage polarization and pyroptosis have both been implicated in LPS-induced ALI (6), while inhibition of the C3a receptor attenuates lung injury by suppressing pyroptosis in pulmonary endothelial cells (7). Although macrophage polarization and pyroptosis are recognized as critical contributors to inflammatory tissue injury, the mechanistic interplay between these processes during LPS-induced ALI remains incompletely understood.

Mesenchymal stem cells (MSCs) possess potent self-renewal capacity, immunomodulatory activity, and multilineage differentiation potential, making them attractive candidates for regenerative therapy (8). Among them, adipose-derived mesenchymal stem cells (ADMSCs) are particularly appealing because of their abundance, accessibility, and therapeutic efficacy in inflammatory diseases, including sepsis (9). Experimental studies have shown that ADMSCs attenuate LPS-induced ALI while improving gut microbiota dysbiosis in septic animals (10). More recently, increasing attention has shifted toward the therapeutic effects of exosomes released by MSCs, which reproduce many of the beneficial paracrine actions of their parental cells while avoiding several limitations associated with cell-based therapy. ADMSC-derived exosomes (ADMSC-Exo) have been shown to alleviate sepsis-induced inflammation by reducing inflammatory cytokine production and tissue injury (11). For example, Wang et al. demonstrated that ADMSC-Exo ameliorated sepsis-induced lung injury by suppressing macrophage-derived interleukin-27 (IL-27) production (12). In addition, ADSC-Exos inhibit NLRP3 inflammasome activation and pyroptosis while promoting macrophage polarization from the pro-inflammatory M1 phenotype toward the anti-inflammatory M2 phenotype, thereby alleviating ALI (13). Similarly, depletion of angiopoietin-like 4 attenuates LPS-induced ALI through inhibition of NF-κB activation, accompanied by reduced M1 macrophage polarization and NLRP3 inflammasome-mediated pyroptosis (14). Despite these observations, the molecular mechanisms by which ADMSC-Exo regulate NLRP3 inflammasome-dependent macrophage polarization and pyroptosis during LPS-induced ALI remain largely undefined.

Transforming growth factor beta receptor II (TGFBR2) has recently emerged as an important regulator of inflammatory injury. TGFBR2 expression is markedly increased in patients with sepsis and in LPS-stimulated human bronchial epithelial BEAS-2B cells, whereas its silencing promotes cell viability and attenuates inflammatory responses (15). In addition, ADMSC-Exo carrying miR-671 alleviates myocardial infarction by targeting the TGFBR2/Smad2 signaling pathway (16), suggesting that exosomal microRNAs may regulate TGFBR2 signaling in inflammatory diseases. However, whether ADMSC-Exo protects against LPS-induced ALI through modulation of TGFBR2 and the underlying molecular mechanisms remain to be further elucidated.

To identify potential upstream regulators of TGFBR2, microRNAs (miRNAs) enriched in mouse-derived exosomes were retrieved from the ExoCarta database, candidate miRNAs targeting TGFBR2 were predicted using multiple bioinformatic databases, and 27 differentially expressed miRNAs associated with sepsis were collected from published studies (17). Integration of these datasets using the Jvenn online platform identified miR-19b-3p as a shared candidate enriched in ADMSC-Exo, with predicted binding to TGFBR2. Previous studies have shown that miR-19b-3p is downregulated in patients with sepsis and that its overexpression alleviates LPS-induced inflammatory responses (18). However, whether ADMSC-Exo exert their protective effects against LPS-induced ALI by delivering miR-19b-3p to regulate TGFBR2 expression has not yet been investigated.

Canonical transforming growth factor-β (TGF-β) signaling is initiated through activation of type I and type II serine/threonine kinase receptors (TβRI and TβRII). Following receptor activation, Smad2 and Smad3 are phosphorylated and subsequently form a transcriptional complex with mothers against decapentaplegic homolog 4 (Smad4) (19), which translocates into the nucleus to regulate target gene transcription (20). Consistent with its role in inflammatory signaling, Smad4 expression is increased in LPS-stimulated human pulmonary microvascular endothelial cells (21), whereas hydrogen treatment attenuates septic encephalopathy in mice by reducing Smad4 expression (22). Furthermore, TGFBR2 activates downstream TGF-β/Smad signaling (23), and upregulation of TGFBR2 has been shown to promote LPS-induced sepsis progression through activation of the TGFBR2/Smad pathway (24). As the common transcriptional mediator of this pathway, Smad4 directly regulates the transcription of multiple downstream genes (25). Bioinformatic analysis using the JASPAR database further predicted putative Smad4-binding sites within the NLRP3 promoter, suggesting a potential transcriptional link between TGFBR2/Smad signaling and NLRP3 inflammasome activation.

Based on these findings, we hypothesized that ADMSC-Exo deliver miR-19b-3p to suppress TGFBR2 expression, thereby inhibiting Smad4-mediated transcriptional activation of NLRP3. This signaling cascade may suppress macrophage M1 polarization and pyroptosis, ultimately attenuating LPS-induced acute lung injury. Therefore, the present study aimed to elucidate the role of the miR-19b-3p/TGFBR2/Smad4/NLRP3 signaling axis in the protective effects of ADMSC-Exo and to provide mechanistic insights into exosome-based therapeutic strategies for sepsis-associated ALI.

## 2 Materials and methods

### 2.1 Ethics statement

All animal procedures were approved by the Medical Ethics Committee of Guangdong Provincial People’s Hospital Ganzhou Hospital, Ganzhou Municipal Hospital (Approval no. SKJJ2024001A) and conducted in accordance with institutional guidelines for the care and use of laboratory animals. Every effort was made to minimize animal use and alleviate animal suffering throughout the study.

### 2.2 Bioinformatics analysis

The ExoCarta database (http://www.exocarta.org/) was queried to identify miRNAs enriched in mouse-derived exosomes, yielding 1, 455 candidate miRNAs (**Supplementary File 1**). Candidate upstream miRNAs targeting TGFBR2 were predicted using the StarBase (https://rnasysu.com/encori/), miRDB (https://mirdb.org/), and miRWalk (http://mirwalk.umm.uni-heidelberg.de/) databases (**Supplementary Files 2–4**). In addition, 27 sepsis-associated differentially expressed miRNAs were collected from a previous study (17) (**Supplementary File 5**). Candidate miRNAs were identified by intersecting these datasets using the Jvenn online website (https://jvenn.toulouse.inra.fr/app/example.html), which identified miR-19b-3p as a potential ADMSC-derived exosomal miRNA targeting TGFBR2 (**Supplementary File 6**).

### 2.3 Culture and characterization of ADMSCs

Mouse ADMSCs (CP-M138, Procell, Wuhan, Hubei, China) were cultured in ADMSC complete medium (CM-M138, Procell) at 37 °C in a humidified incubator containing 5% CO_2_.

The immunophenotype of ADMSCs was characterized by flow cytometry. Cells were harvested by trypsinization, centrifuged at 800 × g for 6 min, washed with phosphate-buffered saline (PBS), and incubated with fluorescein isothiocyanate (FITC)-conjugated antibodies CD29-FITC (G0571, Selleck), CD34-FITC (G0121, Selleck), CD44-FITC (ab25064, Abcam), and CD45-FITC (ab210225, Abcam). Subsequent flow cytometric detection was performed using a FACSCalibur flow cytometer (Becton Dickinson, USA) equipped with FlowJo software (version vX 0.7) to identify the phenotypic characteristics of ADMSCs.

The multilineage differentiation potential of ADMSCs was further evaluated as previously described (26, 27). For osteogenic differentiation, 3 × 10^4^ cells were seeded into six-well plates and cultured in osteogenic induction medium for 4 weeks, followed by fixation with 4% paraformaldehyde (PFA) for 20 min and staining with 1% Alizarin Red S solution (G1450, Solarbio).

For adipogenic differentiation, 2 × 10^4^ cells were cultured in adipogenic induction medium A for 3 days, followed by medium B for 1 day, with the induction cycle repeated three times. Cells were then fixed with 4% PFA for 30 min and stained with Oil Red O (G1262, Solarbio).

For chondrogenic differentiation, 2.5 × 10^5^ cells were cultivated as pellet cultures in chondrogenic induction medium. After 21 days, pellets were fixed with 4% PFA, embedded in paraffin, sectioned, and stained with Alcian blue (G1562, Solarbio). Stained samples were washed with PBS and observed under an Olympus microscope (Tokyo, Japan).

### 2.4 Isolation, characterization, and grouping of ADMSC-derived exosomes

Exosomes were isolated from ADMSC-conditioned medium by differential ultracentrifugation as previously described (28). Briefly, ADMSCs at 80 – 90% confluence (passages 3 – 5) were washed three times with PBS and cultured for 48 h in Dulbecco’s Modified Eagle Medium/Ham’s F-12 supplemented with 10% exosome-depleted fetal bovine serum (Umibio, Shanghai, China). Conditioned medium was sequentially centrifuged at 300 × g for 20 min, 2, 000 × g for 30 min, and 12, 000 × g for 30 min at 4 °C to remove cells and debris. The supernatant was filtered through a 0.22-µm membrane filter (Millipore, Billerica, MA, USA) and ultracentrifuged at 100, 000 × g using a Beckman Coulter Optima L-80 XP ultracentrifuge to pellet exosomes. The exosome pellet was resuspended in 100 – 200 μL PBS solution and stored at -80 °C until further use (29).

Exosome morphology was examined by transmission electron microscopy (TEM; Tecnai G2, FEI, USA), while particle size distribution and concentration were determined by nanoparticle tracking analysis (NTA; Malvern Panalytical, Worcestershire, UK). Western blot analysis was performed to detect the exosomal markers, including CD9 (1:1000, ab236630, Abcam), CD81 (1:1000, ab109201, Abcam), and Tsg101 (1:1000, ab133586, Abcam), together with the negative control marker Calnexin (1:1000, ab241154, Abcam). Culture supernatants from ADMSCs treated with GW4869 (10 μM), an inhibitor of exosome secretion, served as the negative control (GW) (30). Exosomal protein concentration was determined using a bicinchoninic acid (BCA) protein assay kit (P0010, Beyotime, Shanghai, China), and the protein-to-particle ratio was approximately 1 μg per 7.4 × 10^9^ particles. For functional experiments, exosomes were divided into four groups: (1) GW group; (2) Exos group; (3) Exo-antag NC group: exosomes isolated from ADMSCs treated with 1 µM of the antagonist NC for 48  h; (4) Exo-miR antag group: exosomes isolated from ADMSCs treated with 1  µM of the miR-19b-3p antagonist for 48  h (31). For exosome preparation, ADMSCs were transfected with 1 μM antagonist NC or miR-19b-3p antagonist (GENECHEM Co., Ltd., Shanghai, China) for 48 h before exosome isolation.

### 2.5 Experimental animals

Male C57BL/6 mice (8 weeks old, 18-22g) were purchased from Guangzhou Ruige Biotechnology Co., Ltd. (Guangzhou, China). Animals were housed under specific pathogen-free conditions with free access to food and water under a 12-h light/dark cycle at 22–24 °C and 45-55% humidity.

### 2.6 LPS-induced acute lung injury model

Following one week of acclimatization, acute lung injury (ALI) was induced by intraperitoneal injection of lipopolysaccharide (LPS, 10 mg/kg) as previously described (29). For gene manipulation, mice received a tail vein injection of 20 μ adenoviral vectors (1 × 10^7^ particles/μL) expressing Ad-NC, Ad-TGFBR2, Ad-sh-NC, or Ad-sh-TGFBR2 one week before LPS administration (32). One hour after LPS injection, mice were intravenously administered 100 μg ADMSC-derived exosomes (7.4 × 10^9^ particles/μg in 100 μL PBS), Exos-antag NC, Exos-miR-19b-3p antag, or an equal volume of PBS (29). All adenoviral vectors were purchased from GENECHEM (Shanghai, China).

Mice were randomly assigned to ten groups (n = 6 per group): (1) Control group; (2) LPS group; (3) LPS + PBS group: LPS-induced mice received an equal volume of PBS (100 μL) *via* tail vein injection, equivalent to that of ADMSC-Exo; (4) LPS + Exos group: LPS-induced mice received a tail vein injection of ADMSC-Exo; (5) LPS + Exos + Ad-NC group: LPS-induced mice were injected with the Ad-NC adenoviral vector *via* tail vein, followed by an ADMSC-Exo injection; (6) LPS + Exos + Ad-TGFBR2 group: LPS-induced mice underwent an tail vein injection of the adipose-derived transforming growth factor beta receptor II (Ad-TGFBR2) adenoviral vector, followed by ADMSC-Exo; (7) LPS + Exos-antag NC group: LPS-induced mice received Exos-antagonist NC *via* a tail vein injection; (8) LPS + Exos-miR antag group: LPS-induced mice received an Exos-miR-19b-3p antagonist injection *via* tail vein; (9) LPS + Exos-miR antag + Ad-sh-NC group: LPS-induced mice were injected with Exos-miR-19b-3p antagonist after a tail vein injection of Ad-sh-NC; (10) LPS + Exos-miR antag + Ad-sh-TGFBR2 group: LPS-induced mice were subjected to a tail intravenous injection of Ad-sh-TGFBR2, before an Exos-miR-19b-3p antagonist injection.

Twenty-four hours after LPS administration, mice were euthanized under isoflurane anesthesia (33). Briefly, mice were placed in an induction chamber with 5% isoflurane. An appropriate anesthetic plane was achieved within 1 min, confirmed by complete loss of consciousness and disappearance of the righting and pedal reflexes. Mice were immediately removed from the chamber and maintained under anesthesia with 2%–3% isoflurane via a face mask, followed by rapid cervical dislocation to confirm euthanasia (34). Lung tissues were immediately harvested afterwards. The left lung was divided for histological examination and tissue homogenization, whereas the right lung was used for determination of the wet-to-dry (W/D) weight ratio.

Randomization and investigator blinding were implemented throughout the animal study. Mice were allocated to experimental groups using a random number table. Animal allocation, experimental procedures, outcome assessment, and statistical analyses were conducted by independent investigators blinded to group assignments. Mice that died before completion of the experimental protocol were predefined as exclusion criteria; however, no animals died during the study, and all enrolled mice were included in the final analyses.

### 2.7 Hematoxylin and eosin staining

Lung tissues were fixed in 4% paraformaldehyde, dehydrated through graded ethanol, cleared with xylene, embedded in paraffin, sectioned into 5-μm slices, and stained with hematoxylin and eosin (C0105S, Beyotime).

Histopathological changes were examined under a light microscope (Olympus, Tokyo, Japan). Lung injury was evaluated using the blinded scoring system recommended by the American Thoracic Society (35). Briefly, alveolar wall thickening, pulmonary edema, and inflammatory cell infiltration/congestion were each scored on a scale of 0-3 (0 = absent, 1 = mild, 2 = moderate, and 3 = severe), yielding a maximum total score of 9 (36). All histological assessments were performed independently by pathologists blinded to the experimental groups.

### 2.8 Lung wet-to-dry weight ratio

Pulmonary edema was evaluated by measuring the lung wet-to-dry (W/D) weight ratio (37). Briefly, the right lung was excised immediately and weighed to obtain the wet weight (W). Lung tissues were then dried at 65 °C for 72 h to determine the dry weight (D). The W/D ratio was calculated as: W/D ratio = wet weight/dry weight.

### 2.9 Immunofluorescence

Paraffin-embedded lung tissue sections were blocked for 2  h at room temperature with PBS containing 0.3% Triton X-100 (Sigma-Aldrich, St Louis, MO, USA) and 5% bovine serum albumin (Beyotime). Cell slides were fixed with 4% paraformaldehyde (Beyotime) for 15 min. Tissue sections and cell samples were then incubated overnight at 4 °C with primary antibodies against TGFBR2 (1:500, 66636-1-Ig, Proteintech, Wuhan, Hubei, China), GSDMD-N (1:200, YM8489, Immunoway Biotechnology Co., Grapevine, TX, USA), F4/80 (1:12000, 29414-1-AP, Proteintech), inducible nitric oxide synthase (iNOS; 1:1200, 18985-1-AP, Proteintech), and Fizz1 (1: 2000, #500-P214, PeproTech, Rocky Hill, NJ, USA).

Following three washes with PBS, samples were incubated for 1 h at room temperature with FITC-conjugated goat anti-rabbit IgG (1:200, P0186, Beyotime) or Cy3-conjugated goat anti-rabbit IgG (1:200, P0183, Beyotime). Nuclei were counterstained with 4′, 6-diamidino-2-phenylindole (DAPI; P0131, Beyotime), and fluorescent images were captured using an Olympus BX43 fluorescence microscope (Olympus, Tokyo, Japan).

The proportions of TGFBR2-positive macrophages (F4/80^+^TGFBR2^+^), pyroptotic macrophages (F4/80^+^GSDMD-N^+^), M1 macrophages (iNOS^+^), and M2 macrophages (Fizz1^+^) were quantified using Image Pro Plus 6.0 software (Media Cybernetics, Silver Spring, MD, USA).

### 2.10 Cell culture and experimental grouping

RAW 264.7 mouse macrophages (CL-0190, Procell) were cultured in RAW 264.7-specific growth medium (CM-0190, Procell). Primary mouse type II alveolar epithelial cells (CP-M003, Procell) were isolated from pooled lungs of three to five mice from the same batch and cultured in complete mouse type II alveolar epithelial cell culture medium (CM-M003, Procell). Cells were maintained at 37 °C in a humidified incubator containing 5% CO_2_.

To establish the inflammatory model, RAW264.7 cells were stimulated with 1 μg/mL LPS (Sigma-Aldrich) for 24 h (29). RAW264.7 cells were assigned to the following experimental groups: (1) Control group; (2) LPS group; (3) PBS group: cells treated simultaneously with LPS and an equal volume of PBS; (4) Exos group: cells treated simultaneously with LPS and Exos at a final concentration of 10 μg/mL (7.4 × 10^9^ particles/μg) (38); (5) Exos + oe-NC group: oe-NC-delivered cells processed with LPS and Exos; (6) Exos + oe-TGFBR2 group: after oe-TGFBR2 transfection, cells received LPS and Exos treatment; (7) Exo-miR antag NC group: cells co-treated with LPS and Exo-miR antagonist NC; (8) Exo-miR antag group: cells co-treated with LPS and Exo-miR-19b-3p antagonist; (9) Exo-miR antag + si-NC group: following si-NC transfection, cells received treatment with LPS and Exo-miR-19b-3p antagonist; (10) Exo-miR antag + si-TGFBR2 group: following si-TGFBR2 transfection, cells subjected to LPS and Exo-miR-19b-3p antagonist treatment; (11) Exos + oe-Smad4 group: following oe-Smad4 transfection, cells treated with LPS and Exos; (12) Exos + oe-NLRP3 group: after oe-NLRP3 transfection, cells intervened with LPS and Exos.

To evaluate macrophage-mediated epithelial injury, mouse type II alveolar epithelial cells were cultured with conditioned medium collected from each macrophage treatment group. The conditioned medium (CM) groups were as follows: (1) CM-(Control) group; (2) CM-(LPS) group; (3) CM-(GW) group; (4) CM-(Exos) group; (5) CM-(Exos + oe-NC) group; (6) CM-(Exos + oe-TGFBR2) group; (7) CM-(Exos-antag NC) group; (8) CM-(Exos-miR antag) group; (9) CM-(Exos-miR antag + si-NC) group; (10) CM-(Exos-miR antag + si-TGFBR2) group; (11) CM-(Exos + oe-NC) group; (12) CM-(Exos + oe-Smad4) group; (13) CM-(Exos + oe-NLRP3) group.

TGFBR2 overexpression plasmids (oe-TGFBR2), TGFBR2 siRNA (si-TGFBR2), Smad4 overexpression plasmids (oe-Smad4), NLRP3 overexpression plasmids (oe-NLRP3), and their respective negative controls (oe-NC and si-NC) were purchased from GenePharma (Shanghai, China). Transfections were performed using Lipofectamine 2000 (Invitrogen, Carlsbad, CA, USA) according to the manufacturer’s instructions at a final nucleic acid concentration of 50 nM.

### 2.11 Exosome uptake assay

Exosome uptake by RAW 264.7 cells was evaluated using PKH26 fluorescent labeling (Sigma-Aldrich). PKH26-labeled Exos (10 μg/mL) were incubated with LPS-treated RAW264.7 cells. After incubation, cells were fixed with 4% paraformaldehyde for 20 min in the dark, permeabilized with cold methanol, and counterstained with DAPI (Thermo Fisher Scientific). Fluorescent images were acquired using a confocal laser scanning microscope (Olympus, Tokyo, Japan).

### 2.12 Reverse transcription-quantitative polymerase chain reaction

Total RNA was extracted from lung tissue homogenates and RAW 264.7 cells using TRIzol reagent (Invitrogen, Carlsbad, CA, USA). Complementary DNA (cDNA) was synthesized using the PrimeScript RT Reagent Kit (Takara, Dalian, Liaoning, China). Quantitative PCR was performed using SYBR Green II Master Mix (Takara, Tokyo, Japan) on an ABI PRISM 7900 Real-Time PCR System (Applied Biosystems, Foster City, CA, USA). The PCR cycling conditions were as follows: initial denaturation at 95 °C for 10 min, followed by 40 cycles of 95 °C for 10 s, 60 °C for 20 s, and 72 °C for 34 s. GAPDH and U6 served as the internal reference for mRNA and miRNA analyses, respectively. Relative gene expression was calculated using the 2^-ΔΔCt^ method. Primer sequences were synthesized by Sangon Biotech (Shanghai, China) and are listed in **Table 1**.

**Table 1:** Primer sequences of each gene.

Gene	Forward 5’-3’	Reverse 5’-3’
TGFBR2	GTAGCTCTGATGAGTGCAATGAC	CAGATATGGCAACTCCCAGTG
miR-19b-3p	TGTGCAAATCCATGCAAAAC	CTCAACTGGTGTCGTGGAGTC
Smad4	ACTGTGGATGGCTATGTGGAT	CCTTTGCCTATGTGCAACCT
NLRP3	CACCTCTTCTCTGCCTACCTG	AGCTGTAAAATCTCTCGCAGT
GAPDH	CCACCTTCATCTTACCATTTATTATCGC	TTTGATGTTAGTGGGGTCTCG
U6	ATTGGAACGATACAGAGAAGATT	GGAACGCTTCACGAATTTC

### 2.13 Western blot

Total protein was extracted from lung tissue homogenates, RAW 264.7 cells, and lung epithelial cells using RIPA lysis buffer supplemented with protease inhibitors (Solarbio, Beijing, China). Protein concentrations were determined using a bicinchoninic acid (BCA) protein assay kit (Beyotime, Shanghai, China) (39). Equal amounts of protein were separated by 10% sodium dodecyl sulfate–polyacrylamide gel electrophoresis (SDS-PAGE) and subsequently transferred onto polyvinylidene fluoride (PVDF) membranes (Millipore, Billerica, MA, USA). The membranes were blocked with 5% skim milk prepared in Tris-buffered saline containing 0.1% Tween-20 (TBST) for 1 h at room temperature to prevent nonspecific binding. After blocking, membranes were incubated overnight at 4 °C with primary antibodies against TGFBR2 (1:1000, ab259360), Cleaved Caspase-1 (1:1000, 4199, CST), GSDMD-N (1:1000, ab215203), NLRP3 (1:1000, ab263899), Smad4 (1:5000, ab40759), B-cell lymphoma-2 (Bcl-2) (1:2000, ab182858), Bcl-2-associated X (Bax) (1:1000, ab182733), Cleaved Caspase-3 (1:1000, PA5114687, PeproTech), and GAPDH (1:10000, ab9485). Following incubation with primary antibodies, membranes were washed with horseradish peroxidase (HRP)-conjugated goat anti-rabbit IgG secondary antibody (1:2000, ab48386) for 1 h at room temperature. Protein bands were visualized using an enhanced chemiluminescence (ECL) detection system (Millipore) and quantified using ImageJ software (version 1.48, National Institutes of Health, Bethesda, MD, USA). GAPDH was used as the endogenous loading control.

### 2.14 Dual-luciferase reporter assay

Potential binding sites between miR-19b-3p and TGFBR2 were predicted using the StarBase database. Wild-type (WT) and mutant-type (MUT) fragments of the TGFBR2 3′-untranslated region (3′-UTR) containing the predicted miR-19b-3p binding sites were synthesized by GenePharma (Shanghai, China) and cloned into the pmir-GLO luciferase reporter vector to generate TGFBR2-WT and TGFBR2-MUT reporter plasmids. Furthermore, potential binding sites between Smad4 and the NLRP3 promoter region were predicted using the JASPAR database (https://jaspar.elixir.no/). The predicted Smad4-binding sequence within the NLRP3 promoter and its corresponding mutant sequence were amplified and inserted into the pmir-GLO luciferase reporter vector (GenePharma) to generate NLRP3-WT and NLRP3-MUT plasmids. RAW 264.7 cells were co-transfected with TGFBR2-WT/TGFBR2-MUT plasmids and mimics NC/miR-19b-3p mimics, or with NLRP3-WT/NLRP3-MUT plasmids and oe-NC/oe-Smad4 using Lipofectamine™ 2000 reagent (Invitrogen, Carlsbad, CA, USA) according to the manufacturer’s instructions. After 48 h of transfection, luciferase activity was determined using a dual-luciferase reporter assay kit (Promega, Madison, WI, USA).

### 2.15 Chromatin immunoprecipitation assay

Chromatin immunoprecipitation (ChIP) was performed to verify the binding of Smad4 to the NLRP3 promoter region. Briefly, 1 × 10^8^ RAW 264.7 cells were fixed with 1% formaldehyde (Solarbio, Beijing, China) and sonicated on ice using a 2-mm probe at 40% amplitude and 90% duty cycle for 8 min (10 s on/15 s off cycles) to shear chromatin into suitable fragments. Following sonication, chromatin samples (0.5 mL) were centrifuged to remove debris. A total of 150 μL of fragmented chromatin was diluted 9-fold and incubated with 60 μL of protein G agarose beads (Thermo Fisher Scientific, Waltham, MA, USA) at 4 °C for 4 h with rotation. After centrifugation at 1100 g for 5 min, the supernatant was collected, and 5% of the sample was reserved as the input control. The remaining chromatin samples were incubated overnight at 4 °C with anti-Smad4 antibody (1:1000, ab236321, Abcam) or control IgG antibody (5 µg/mL, #2729, Cell Signaling Technology, Danvers, MA, USA). Protein-DNA complexes were subsequently captured using 60 μL of Pierce™ Protein A/G magnetic beads (Thermo Fisher Scientific) at 4 °C for 6 h. After reverse cross-linking and proteinase K (Thermo Fisher Scientific), the immunoprecipitated DNA precipitates were purified and diluted in 100 μL of 0.2 M glycine solution. The enrichment of target DNA fragments was quantified by RT-qPCR using an ABI PRISM 7900 sequence detection system with SYBR Green II (TaKaRa Biotechnology, Tokyo, Japan). The amplification conditions were as follows: initial denaturation at 95 °C for 5 min, followed by 40 cycles of 95 °C for 15 s, 60 °C for 20 s, and 72 °C for 35 s. The primer sequences used for amplification of the NLRP3 promoter region were as follows: NLRP3 Forward: 5’-GCCACTCTTGTGTCCTCAGA-3’, NLRP3 Reverse: 5’-GCAACACAGCAAGAGGCAAA-3’.

### 2.16 Flow cytometry

The proportions of M1 and M2 macrophages in mouse lung tissue and RAW 264.7 cells were analyzed by flow cytometry. Mouse lung tissues were dissociated into single-cell suspensions. Lung-derived single-cell suspensions or RAW 264.7 cells were adjusted to a density of 1 × 10^6^ cells/mL in PBS and incubated with anti-CD86-FITC (2 µg/mL, ab234237, Abcam), anti-CD206-PE (2.5 µg/mL, 98031-1-RR, Proteintech), and anti-F4/80-PE (1:100, ab237335, Abcam), anti-CD86-APC (0.5 µg/mL, ab326464, Abcam), anti-CD206-APC (0.2 mg/mL, G4068, Selleck) antibodies at 4 °C in the dark for 30 min. Samples were analyzed using a FACSCalibur flow cytometer (Becton Dickinson, USA), and data were analyzed with FlowJo software (version X 0.7). Cells were first gated based on forward scatter area (FSC-A) and side scatter area (SSC-A) to exclude debris and identify the main leukocyte population (P1). Doublets were then excluded by gating on FSC-A against FSC-height (FSC-H) to identify single cells (P2). Viable cells were identified as Fixable Viability Stain (FVS) (557) (L34979, Thermo Fisher Scientific, Excitation/Emission: 479/557 nm) (P3). The proportions of positive CD86-FITC^+^ (M1 macrophages) and CD206-PE^+^ (M2 macrophages) in RAW 264.7 cells were quantified accordingly. For lung tissue homogenate-derived single-cell suspensions, macrophages were initially gated based on F4/80-PE fluorescence. Positive expression of CD86-APC and CD206-APC was then evaluated, and the proportions of F4/80-PE^+^CD86-APC^+^ (M1 macrophages) and F4/80-PE^+^CD206-APC^+^ (M2 macrophages) were subsequently calculated.

Cell apoptosis was assessed by flow cytometry. Pulmonary epithelial cells were collected, adjusted to 1 × 10^6^ cells/mL with PBS, and centrifuged. Subsequent staining was carried out following the manufacturer’s protocol for the Annexin V-FITC Apoptosis Detection Kit (BD Bioscience, Franklin Lakes, NJ, USA). Briefly, the cell pellet was resuspended in binding buffer, incubated with 1.0 mL propidium iodide (PI) for 15 min at 4 °C in the dark. Samples were analyzed using a FACSCalibur flow cytometer (Becton Dickinson, USA).

### 2.17 Enzyme-linked immunosorbent assay

The concentrations of IL-1β (CB10173-Mu), IL-18 (CB10172-Mu), TNF-α (CB10851-Mu), and IL-10 (CB10161-Mu) in lung tissue homogenates and RAW 264.7 cell supernatants were determined using commercially available ELISA kits (Keaibo Biotechnology Co., Ltd., Shanghai, China) according to the manufacturer’s instructions.

### 2.18 Lactate dehydrogenase release assay

Cell culture supernatants were collected, and LDH release was quantified using an LDH Release Assay Kit (C0017, Beyotime) according to the manufacturer’s instructions. Absorbance was measured at 490 nm using a microplate reader.

### 2.19 Caspase-1 activity assay

Caspase-1 activity in lung tissues and RAW 264.7 cells was measured using a Caspase-1 activity assay kit (C1101, Beyotime) following the manufacturer’s protocol. Briefly, tissue samples and cells were lysed at 4 °C, and the resulting supernatants were incubated with the caspase-1 substrate Ac-YVAD-pNA at 37 °C for 2  h. The released p-nitroaniline (pNA) was quantified by measuring absorbance at 405 nm using a SpectraMax^®^ iD5 Multi-Mode Microplate Reader (Molecular Devices, Sunnyvale, CA, USA).

### 2.20 Statistical analysis

Statistical analyses were performed using SPSS 21.0 (SPSS, Inc., Chicago, IL, USA). Data normality was assessed using the Shapiro-Wilk test. Normally distributed data are presented as mean ± standard deviation (SD). Comparisons between two groups were performed using the independent-samples *t*-test, whereas comparisons among multiple groups were conducted using one-way analysis of variance (ANOVA), followed by Tukey’s multiple comparison test. All statistical tests were two-tailed, and a *p* < 0.05 was considered statistically significant.

## 3 Results

### 3.1 ADMSC-Exo attenuated LPS-induced ALI in mice by downregulating TGFBR2

ADMSCs were first cultured *in vitro*, and exosomes were subsequently isolated and characterized. Cultured ADMSCs exhibited a typical spindle-shaped morphology with a whirlpool-like growth pattern (**Figure 1**). Flow cytometric analysis confirmed positive expression of the mesenchymal stem cell markers CD29 and CD44 and negative expression of the hematopoietic markers CD34 and CD45 (**Figure 1**). Meanwhile, ADMSCs retained their multilineage differentiation potential, as demonstrated by successful osteogenic, adipogenic, and chondrogenic differentiation (**Figure 1C**).

**Figure 1:**
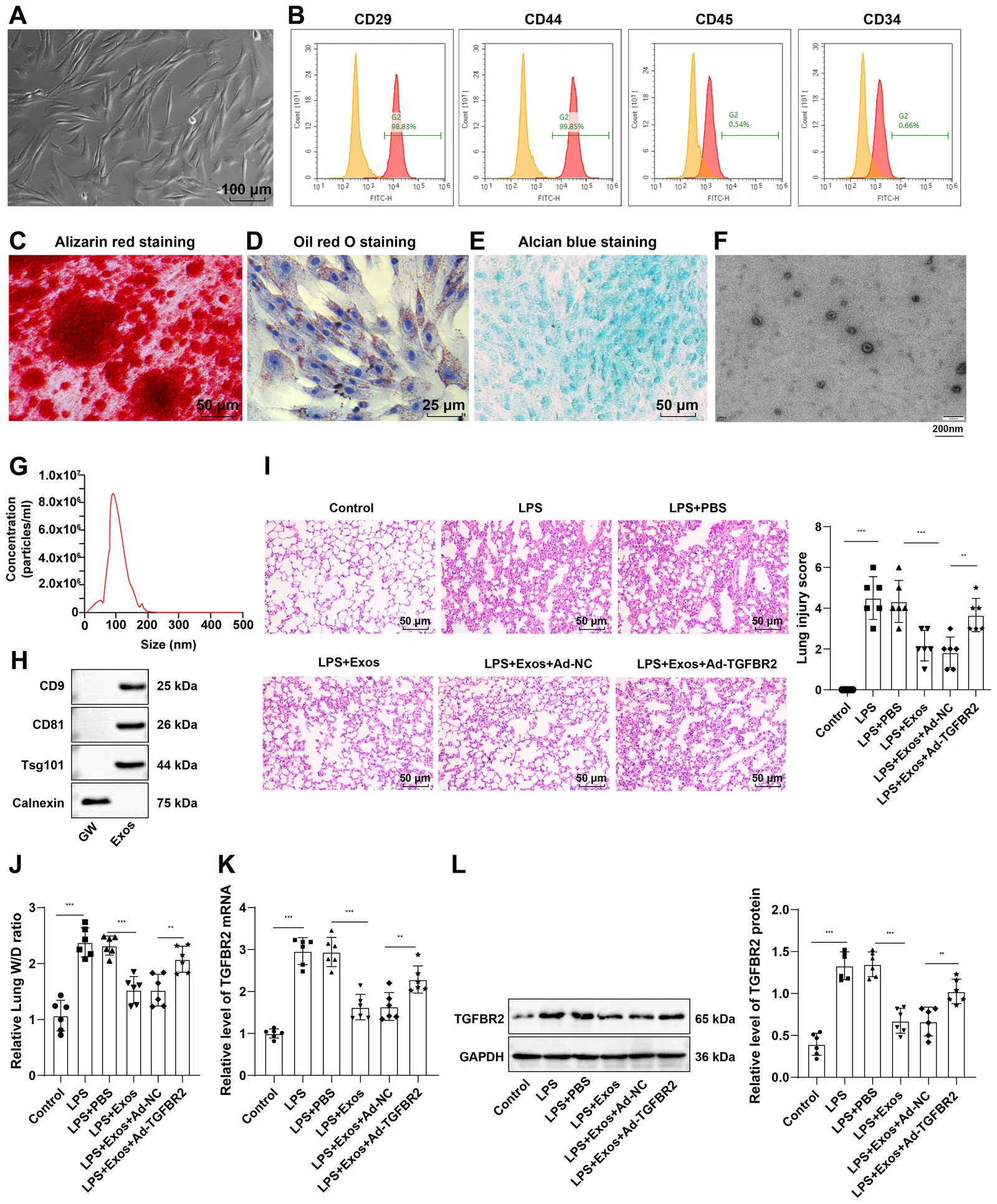
ADMSC-Exo attenuated LPS-induced ALI in mice by downregulating TGFBR2. ADMSCs were cultured *in vitro*, and Exos were isolated and extracted by ultracentrifugation. **(A)** The morphological characteristics of ADMSCs were observed under an inverted microscope. **(B)** The positive markers (CD29 and CD44) and negative markers (CD45 and CD34) of ADMSCs were detected by flow cytometry. **(C–E)** Alizarin red, oil red O, and Alcian blue staining were used to evaluate the differentiation ability of ADMSCs into osteoblast-like, fat-like or chondrocyte-like cells. **(F)** The morphology of ADMSC-Exo was observed by TEM. **(G)** NTA was used to analyze the particle size distribution of ADMSC-Exo; **(H)** Western blot was utilized to detect the expression of CD9, CD81, Tsg101, and Calnexin; **(I)** The pathological changes of lung tissue was evaluated by H&E staining and rated through the lung injury score; **(J)** Determination of pulmonary W/D ratio; **(K)** RT-qPCR and **(L)** western blot were used to measure TGFBR2 expression. n = 6, the data were expressed by mean ± standard deviation, and one-way ANOVA was used for comparison among multiple groups of data, followed by Tukey’s multiple comparisons test. **p < 0.01, ***p < 0.001. ADMSC-Exo, adipose-derived mesenchymal stem cells-exosomes; LPS, lipopolysaccharide; ALI, acute lung injury; TGFBR2, transforming growth factor beta receptor II; RT-qPCR, reverse transcription-quantitative polymerase chain reaction; TEM, transmission electron microscopy; NTA, nanoparticle tracking analysis; H&E, hematoxylin and eosin; W/D, wet/dry.

Transmission electron microscopy revealed that the isolated exosomes displayed the characteristic spherical or cup-shaped morphology enclosed by a lipid bilayer (**Figure 1**). Nanoparticle tracking analysis showed that the vesicles were predominantly distributed around 100 nm in diameter, with a concentration of 8.6 × 10^6^ particles/mL (**Figure 1**). Western blot analysis confirmed the expression of the exosomal markers CD9, CD81, and Tsg101, whereas the endoplasmic reticulum marker Calnexin was absent (**Figure 1**). This confirmed the successful isolation and characterization of ADMSC-derived exosomes (ADMSC-Exo).

To investigate their therapeutic effects, an LPS-induced ALI mouse model was established and treated with ADMSC-Exo. Compared with the Control group, LPS administration caused marked inflammatory cell infiltration, disruption of alveolar architecture, and significant increases in both the lung injury score and lung wet-to-dry (W/D) weight ratio (*p* < 0.01; **Figure 1I**). Treatment with ADMSC-Exo markedly preserved alveolar structure and significantly reduced both the lung injury score and W/D ratio (both *p* < 0.01; **Figure 1I**).

Previous studies have shown that TGFBR2 is aberrantly upregulated in sepsis and contributes to inflammatory injury (15). Consistent with these observations, TGFBR2 expression was significantly increased in the lungs of LPS-treated mice but was markedly reduced following ADMSC-Exo treatment (both *p* < 0.01; **Figure 1K**). To determine whether TGFBR2 mediated the protective effects of ADMSC-Exo, mice receiving LPS and ADMSC-Exo were additionally administered Ad-TGFBR2. Compared with the LPS + Exos + Ad-NC group, the Ad-TGFBR2 group significantly restored TGFBR2 expression in lung tissue (both *p* < 0.05; **Figure 1K**), accompanied by aggravated inflammatory cell infiltration, more severe alveolar structural damage, and significant increases in both the lung injury score and W/D ratio (both *p* < 0.01; **Figure 1I**). Collectively, these findings suggest that ADMSC-Exo alleviate LPS-induced ALI, at least in part, through suppression of TGFBR2 expression.

### 3.2 ADMSC-exo suppressed macrophage M1 polarization and pyroptosis by delivering miR-19b-3p to inhibit TGFBR2

Previous studies have suggested that macrophage pyroptosis is closely associated with M1 polarization during LPS-induced ALI (40). Flow cytometric analysis demonstrated that LPS treatment significantly increased the proportion of M1 macrophages, as evidenced by elevated F4/80^+^CD86^+^, F4/80^+^iNOS^+^, and TNF-α levels (all *p* < 0.01), while reducing M2 polarization, indicated by decreased F4/80^+^CD206^+^, F4/80^+^Fizz1^+^, and IL-10 expression (all *p* < 0.01) (**Figures 2A–F**). ADMSC-Exo treatment markedly suppressed M1 macrophage polarization while promoting M2 polarization (all *p* < 0.01; **Figures 2A–F**). However, TGFBR2 overexpression largely abolished these effects, resulting in enhanced M1 polarization and reduced M2 polarization compared with the LPS + Exos + Ad-NC group (all *p* < 0.01; **Figures 2A–F**).

**Figure 2:**
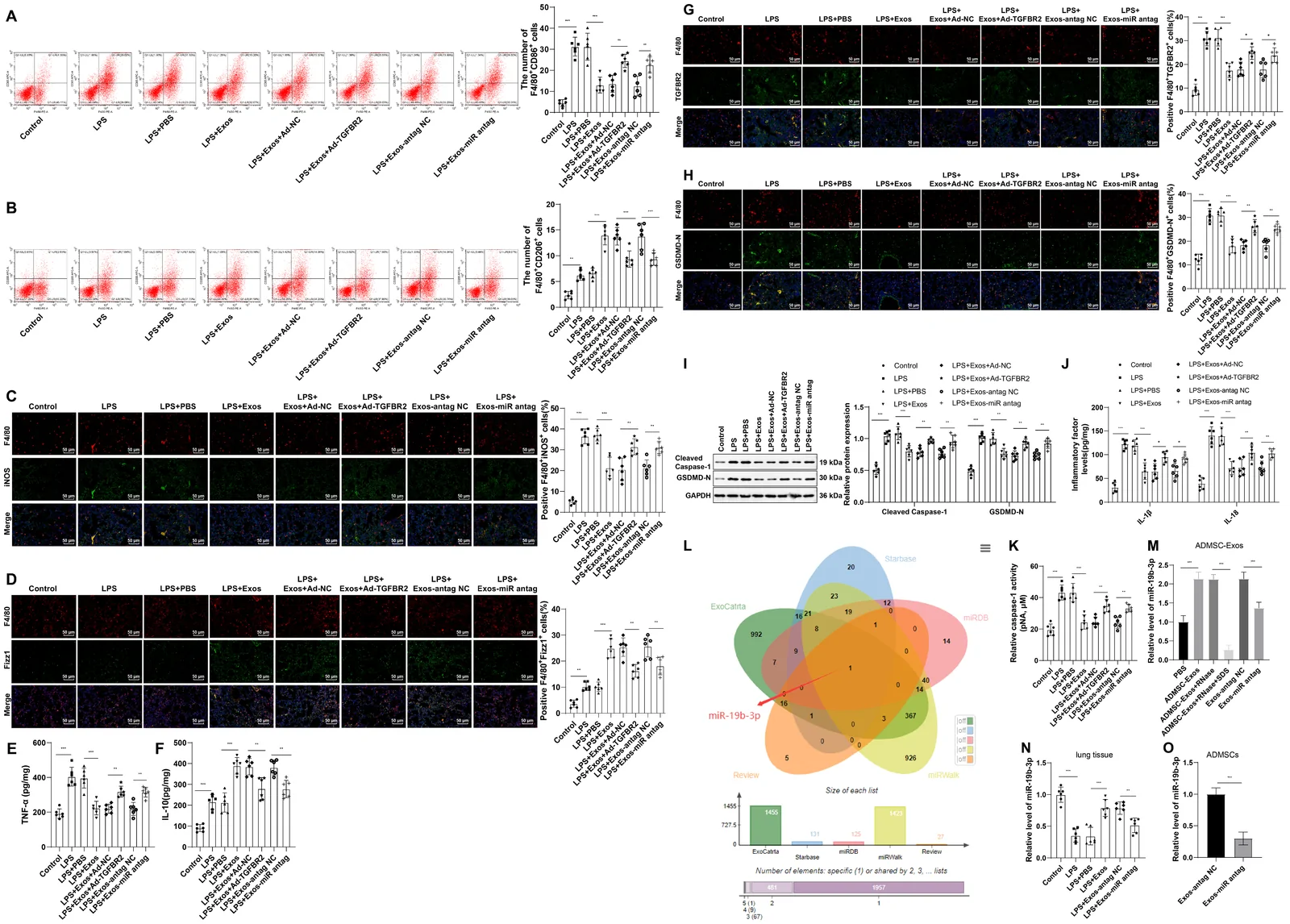
ADMSC-Exo suppressed macrophage M1 polarization and pyroptosis by downregulating TGFBR2 through miR-19b-3p. **(A, B)** Flow cytometry was used to detect the numbers of F4/80^+^CD86^+^(M1 polarization) and F4/80^+^CD206^+^(M2 polarization) cells in lung tissues, n = 6; **(C, D)** Quantification of F4/80^+^iNOS^+^ and F4/80^+^Fizz1^+^ cells in mouse lung tissue by immunofluorescence, n = 6; **(E, F)** Levels of TNF-α and IL-10 in mouse lung tissue measured by ELISA, n = 6; **(G, H)** Immunofluorescence detection of the numbers of F4/80^+^TGFBR2^+^ and F4/80^+^GSDMD-N^+^ cells in macrophages of mouse lung tissue, n = 6; **(I)** Western blot to detect the expression of Cleaved Caspase-1 and GSDMD-N in lung tissues of mice, n = 6; **(J)** ELISA to determine the levels of IL-1β and IL-18 in lung tissues of mice, n = 6; **(K)** Caspase-1 activity in mouse lung tissue measured by ELISA; **(L)** The jvenn online website showing the intersection of miRNAs from ExoCarta, StarBase, miRDB, miRWalk databases, and literature reviews; **(M–O)** RT-qPCR to determine the levels of miR-19b-3p in ADMSC Exos (n = 3), ADMSC cells (n = 3), and lung tissues (n = 6). Data were expressed as mean ± standard deviation. One-way ANOVA was used for comparisons between multiple sets of data, and the Tukey’s multiple comparison test was used for *post hoc* tests. *p < 0.05, **p < 0.01, ***p < 0.001. ADMSC-Exo, adipose-derived mesenchymal stem cells-exosomes; RT-qPCR, reverse transcription-quantitative polymerase chain reaction; ELISA, Enzyme-Linked Immunosorbent Assay; ANOVA, analysis of Variance; IL-1β, interleukin-1 beta; IL-18, interleukin-18; TNF-α, tumor Necrosis Factor-alpha; IL-10, interleukin-10.

Immunofluorescence analysis further demonstrated that TGFBR2 expression and macrophage pyroptosis were markedly increased in lung macrophages following LPS stimulation, whereas both were significantly attenuated by ADMSC-Exo treatment (both *p* < 0.01; **Figures 2G, H**). These protective effects were largely reversed by Ad-TGFBR2 administration, which restored both TGFBR2 expression and macrophage pyroptosis (both *p* < 0.01; **Figures 2G, H**). Consistently, Western blot and ELISA analyses showed that LPS markedly increased the expression of GSDMD-N and Cleaved Caspase-1, together with the pro-inflammatory cytokines inflammatory cytokines IL-1β and IL-18. ADMSC-Exo significantly reduced all of these pyroptosis-associated markers, whereas Ad-TGFBR2 overexpression partially restored their expression (all *p* < 0.01; **Figures 2I–K**).

To investigate how ADMSC-Exo regulates TGFBR2, bioinformatic analyses identified miR-19b-3p as a candidate exosomal miRNA (**Figure 2L**). RT-qPCR demonstrated significantly higher miR-19b-3p expression in the ADMSC-Exo group than in the GW-treated control group (*p* < 0.01). RNase treatment alone did not affect exosomal miR-19b-3p levels (*p* > 0.05), whereas combined RNase and SDS treatment significantly reduced miR-19b-3p expression (*p* < 0.01; **Figure 2M**), indicating that miR-19b-3p was encapsulated within exosomes.

Compared with the Control group, miR-19b-3p expression in lung tissues was significantly decreased following LPS administration but was restored after ADMSC-Exo treatment (*p* < 0.05; **Figure 2N**). To further verify its role, ADMSCs were transfected with a miR-19b-3p antagonist before exosome isolation, resulting in efficient knockdown of miR-19b-3p in both ADMSCs (*p* < 0.001; **Figure 2O**) and the corresponding exosomes (*p* < 0.01; **Figure 2M**). Consequently, mice receiving miR-19b-3p-deficient exosomes exhibited significantly lower pulmonary miR-19b-3p levels than those treated with control exosomes (*p* < 0.01; **Figure 2N**). This was accompanied by increased TGFBR2 expression in macrophages (*p* < 0.05; **Figure 2G**), enhanced M1 macrophage polarization, suppressed M2 polarization (both *p* < 0.05; **Figures 2A–F**), and aggravated macrophage pyroptosis (all *p* < 0.05; **Figures 2G–K**).

Collectively, these findings demonstrate that ADMSC-Exo suppresses macrophage M1 polarization and pyroptosis by delivering miR-19b-3p, thereby downregulating TGFBR2.

### 3.3 ADMSC-exo inhibited LPS-induced macrophage M1 polarization and pyroptosis by suppressing TGFBR2, thereby reducing lung epithelial cell apoptosis

To further investigate the underlying mechanism, RAW 264.7 macrophages were stimulated with LPS and subsequently treated with ADMSC-Exo for 24 h. LPS stimulation significantly increased TGFBR2 mRNA and protein expression in RAW 264.7 cells (all *p* < 0.05; **Figure 3A**). In parallel, the proportion of CD86-positive cells was markedly increased (*p* < 0.05), whereas no significant change was observed in CD206-positive cells (*p* > 0.05; **Figure 3C**). LPS exposure also increased LDH release (*p* < 0.05; **Figure 3**), elevated the expression of Cleaved Caspase-1, GSDMD-N, IL-1β, and IL-18 (all *p* < 0.05; **Figure 3G**), and enhanced Caspase-1 activity (*p* < 0.05; **Figure 3**), indicating macrophage pyroptosis.

**Figure 3:**
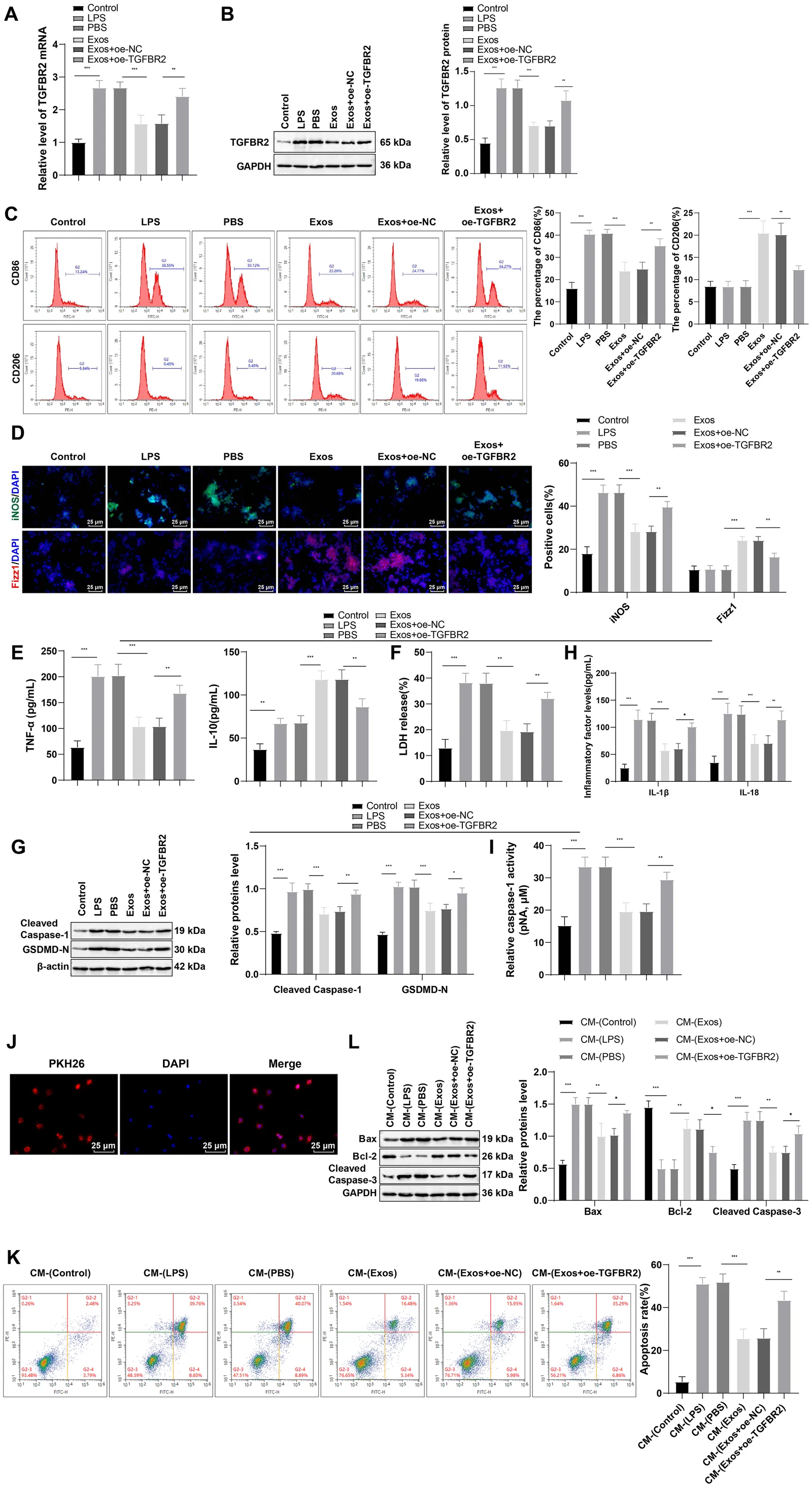
ADMSC-Exo inhibited LPS-induced macrophage M1 polarization and pyroptosis by modulating TGFBR2, thereby reducing lung epithelial cell apoptosis. **(A, B)** RT-qPCR and western blot analyses of TGFBR2 expression in RAW 264.7 cells; **(C)** Flow cytometric analyses of CD86 and CD206 proportions in RAW 264.7 cells; **(D)** Immunofluorescence detection of iNOS and Fizz1 expression in RAW 264.7 cells; **(E)** ELISA determination of TNF-α and IL-10 levels in RAW 264.7 cells; **(F)** Measurement of LDH released from RAW 264.7 cells using an LDH release assay kit; **(G)** Western blot analyses of Cleaved Caspase-1 and GSDMD-N levels in RAW 264.7 cells; **(H, I)** Levels of IL-1β and IL-18, as well as caspase-1 activity, in RAW 264.7 cells measured by ELISA; **(J)** Confocal microscopy to visualize uptake of ADMSC-Exo by RAW 264.7 cells; **(K)** Flow cytometry to analyze apoptosis of lung epithelial cells; **(L)** Western blot analyses of Bax, Bcl-2, and Cleaved Caspase-3 expression in lung epithelial cells. Cell experiments were repeated three times. Data of the normal distribution were expressed as mean ± standard deviation. One-way ANOVA was used for multi-group data comparisons. Tukey’s multiple comparison test was used for *post hoc* tests. **p < 0.01, ***p < 0.001. ADMSC-Exo, adipose-derived mesenchymal stem cells-exosomes; RT-qPCR, reverse transcription-quantitative polymerase chain reaction; ELISA, Enzyme-Linked Immunosorbent Assay; ANOVA, analysis of Variance; IL-1β, interleukin-1 beta; IL-18, interleukin-18; TNF-α, tumor Necrosis Factor-alpha; IL-10, interleukin-10; LDH, lactate dehydrogenase.

Immunofluorescence analysis confirmed efficient uptake of ADMSC-Exo by RAW 264.7 cells (**Figure 3**). Compared with LPS treatment alone, co-treatment with LPS and ADMSC-Exo significantly reduced TGFBR2 expression (both *p* < 0.05; **Figure 3A**), decreased the proportion of CD86-positive cells, increased CD206-positive cells (both *p* < 0.05; **Figure 3C**), and attenuated macrophage pyroptosis, as evidenced by reduced LDH release, decreased expression of pyroptosis-related proteins and inflammatory cytokines, and lower caspase-1 activity (all *p* < 0.05; **Figure 3F**). However, these protective effects were largely abolished by TGFBR2 overexpression, as oe-TGFBR2 restored M1 polarization and pyroptotic responses in ADMSC-Exo-treated macrophages (all *p* < 0.05; **Figure 3A**).

To determine whether macrophage modulation affected epithelial cell injury, conditioned medium (CM) collected from RAW 264.7 cells was applied to primary mouse lung epithelial cells. Compared with the CM-(Control) group, conditioned medium from LPS-stimulated macrophages significantly increased epithelial cell apoptosis (*p* < 0.05; **Figure 3**), accompanied by increased Bax and Cleaved Caspase-3 expression and reduced Bcl-2 expression (all *p* < 0.05; **Figure 3**). In contrast, conditioned medium from ADMSC-Exo-treated macrophages significantly attenuated epithelial cell apoptosis, as demonstrated by reduced Bax and Cleaved Caspase-3 expression together with increased Bcl-2 expression (all *p* < 0.05; **Figure 3K**). These protective effects were partially reversed when macrophages overexpressed TGFBR2 before ADMSC-Exo treatment, resulting in increased epithelial cell apoptosis, elevated Bax and Cleaved Caspase-3 levels, and decreased Bcl-2 levels (all *p* < 0.05; **Figure 3K**).

Together, these findings indicate that ADMSC-Exo suppresses LPS-induced macrophage M1 polarization and pyroptosis by downregulating TGFBR2, thereby protecting lung epithelial cells from apoptosis.

### 3.4 ADMSC-Exo transcriptionally suppressed NLRP3 expression by delivering miR-19b-3p to inhibit the TGFBR2/Smad4 signaling pathway

RT-qPCR analysis showed that miR-19b-3p expression was significantly reduced in LPS-stimulated RAW 264.7 cells but was markedly restored following ADMSC-Exo treatment (all *p* < 0.01; **Figure 4**). Bioinformatic analysis predicted a binding site between miR-19b-3p and the 3′-UTR of TGFBR2 (**Figure 4**). Dual-luciferase reporter assays demonstrated that co-transfection of miR-19b-3p mimics with the TGFBR2-WT reporter significantly decreased luciferase activity (*p* < 0.01), whereas no significant change was observed with the TGFBR2-MUT reporter (*p* > 0.05; **Figure 4**), confirming that TGFBR2 is a direct target of miR-19b-3p.

**Figure 4:**
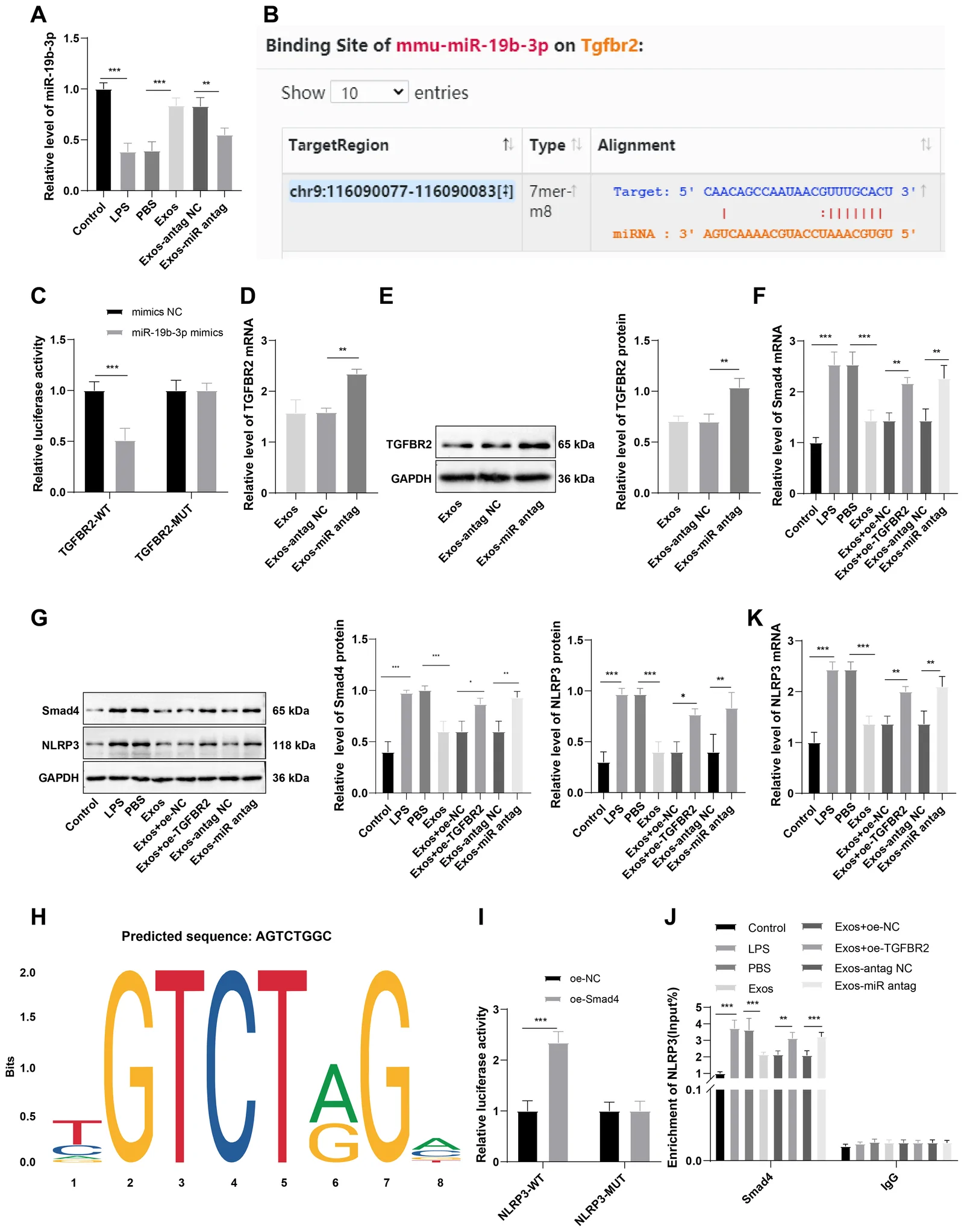
ADMSC-Exo delivered miR-19b-3p to inhibit TGFBR2-mediated Smad4’s transcriptional regulation on NLRP3 expression. **(A)** The RT-qPCR analysis of miR-19b-3p levels in RAW 264.7 cells; **(B)** Predicted binding sites between miR-19b-3p and TGFBR2 based on the StarBase database; **(C, I)** Dual-luciferase reporter assays showing relative luciferase activity; **(D, E)** RT-qPCR and western blot to measure TGFBR2 expression in RAW 264.7 cells; **(F)** RT-qPCR to assess Smad4 mRNA levels in RAW 264.7 cells; **(G)** Western blot analyses of Smad4 and NLRP3 protein levels in RAW 264.7 cells; **(H)** Analyses of target binding sites between Smad4 and NLRP3 promoter region using the JASPAR database (https://jaspar.elixir.no/); **(J)** ChIP assay to detect the enrichment level of Smad4 in the NLRP3 promoter region; **(K)** The RT-qPCR analysis of NLRP3 mRNA levels in RAW 264.7 cells. Cell experiments were repeated three times. Data of the normal distribution were exhibited as mean ± standard deviation. One-way ANOVA was used for data comparison between multiple groups. Tukey’s multiple comparison test was used for *post hoc* tests. *p < 0.05, **p < 0.01, ***p < 0.001. ADMSC-Exo, adipose-derived mesenchymal stem cells-exosomes; ELISA, Enzyme-Linked Immunosorbent Assay; ANOVA, analysis of Variance; RT-qPCR, reverse transcription-quantitative polymerase chain reaction; ChIP, Chromatin immunoprecipitation; NLRP3, nucleotide oligomerization domain-like receptor protein 3.

To further validate this regulatory relationship, RAW 264.7 cells were treated with LPS together with miR-19b-3p-deficient exosomes (Exos-miR antag). Compared with the Exos-miR antag NC group, Exos-miR antag treatment significantly reduced intracellular miR-19b-3p expression (*p* < 0.01; **Figure 4**) and markedly increased TGFBR2 expression (*p* < 0.01; **Figure 4D**).

Previous studies have shown that TGFBR2 positively regulates Smad4 expression (41). Consistent with this finding, both RT-qPCR and Western blot analyses demonstrated that Smad4 expression was significantly increased following LPS stimulation, reduced by ADMSC-Exo treatment, and restored after TGFBR2 overexpression. Likewise, Exos-miR antag treatment significantly increased Smad4 expression compared with Exos-antag NC treatment (all *p* < 0.05; **Figure 4D**).

Bioinformatic analysis using the JASPAR database identified putative Smad4-binding sites within the NLRP3 promoter (**Figure 4**). Dual-luciferase reporter and ChIP assays further confirmed that ADMSC-Exo delivered miR-19b-3p to suppress TGFBR2 expression, thereby reducing Smad4 binding to the NLRP3 promoter (*p* < 0.01; **Figure 4G**). Consistently, NLRP3 expression was significantly elevated in LPS-treated RAW 264.7 cells but was markedly reduced following ADMSC-Exo treatment. In contrast, NLRP3 expression was significantly increased in the Exos-miR antag group compared with the Exos-antag NC group (all *p* < 0.05; **Figure 4G**).

Collectively, these findings demonstrate that ADMSC-Exo delivers miR-19b-3p to directly target TGFBR2, thereby suppressing Smad4-dependent transcriptional activation of NLRP3. This mechanism contributes to the inhibitory effects of ADMSC-Exo on macrophage inflammatory activation.

### 3.5 Inhibition of Exos-miR-19b-3p or overexpression of Smad4/NLRP3 partially reversed the inhibitory effects of ADMSC-Exo on macrophage M1 polarization and pyroptosis, thereby promoting lung epithelial cell apoptosis

Compared with the Exos-antag NC group, the Exos-miR antag group exhibited increased CD86-positive macrophages and decreased CD206-positive macrophages (both *p* < 0.05; **Figure 5A**), accompanied by enhanced macrophage pyroptosis (all *p* < 0.05; **Figure 5D**). Furthermore, overexpression of Smad4 or NLRP3 was successfully achieved in LPS- and ADMSC-Exo-treated RAW 264.7 cells (*p* < 0.01; **Figure 5H**). Compared with the Exos + oe-NC group, Smad4 or NLRP3 overexpression resulted in increased CD86-positive macrophages, reduced CD206-positive macrophages (both *p* < 0.05; **Figure 5A**), and aggravated macrophage pyroptosis (all *p* < 0.05; **Figure 5D**).

**Figure 5:**
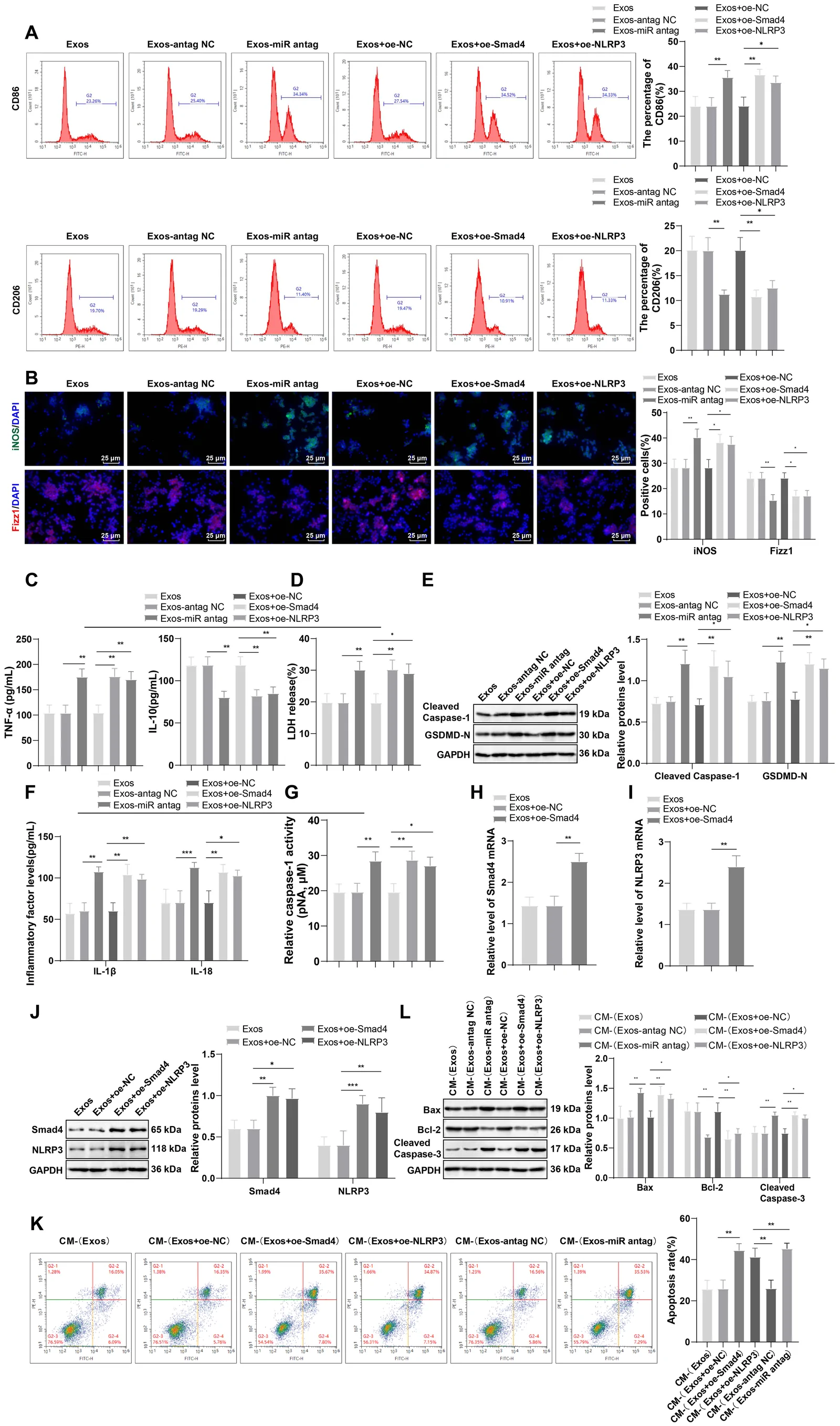
Suppression of Exos-miR-19b-3p or overexpression of Smad4/NLRP3 partially reversed the suppressive effects of ADMSC-Exo on macrophage M1 polarization and pyroptosis, eventually promoting lung epithelial cell apoptosis. **(A)** Flow cytometry to examine the proportions of CD86 and CD206; **(B)** Quantification of iNOS and Fizz1 in RAW 264.7 cells by immunofluorescence; **(C)** Levels of TNF-α and IL-10 in RAW 264.7 cells measured by ELISA; **(D)** LDH release detected using a kit; **(E)** Western blot to measure Cleaved Caspase-1 and GSDMD-N levels in RAW 264.7 cells; **(F, G)** Levels of IL-1β and IL-18, as well as caspase-1 activity, in RAW 264.7 cells measured by ELISA; **(H, I)** RT-qPCR to measure Smad4 and NLRP3 expression in RAW 264.7 cells; **(J)** Western blot analyses of Smad4 and NLRP3 levels in RAW 264.7 cells; **(K)** Flow cytometry to evaluate lung epithelial cell apoptosis; **(L)** Western blot to assess Bax, Bcl-2, and Cleaved Caspase-3 expression in lung epithelial cells. Cell experiments were repeated three times. Data of the normal distribution were presented as mean ± standard deviation. One-way ANOVA was used for data comparisons between multiple groups. Tukey’s multiple comparison test was used for *post hoc* tests. *p < 0.05, **p < 0.01, ***p < 0.001. ADMSC-Exo, adipose-derived mesenchymal stem cells-exosomes; ELISA, Enzyme-Linked Immunosorbent Assay; ANOVA, analysis of Variance; NLRP3, nucleotide oligomerization domain-like receptor protein 3; Smad4, mothers against decapentaplegic homolog 4; ELISA, Enzyme-Linked Immunosorbent Assay; ANOVA, analysis of Variance; IL-1β, interleukin-1 beta; IL-18, interleukin-18; TNF-α, tumor Necrosis Factor-alpha; IL-10, interleukin-10; LDH, lactate dehydrogenase; RT-qPCR, reverse transcription-quantitative polymerase chain reaction.

Subsequently, conditioned medium (CM) collected from differently treated macrophages was used to culture lung epithelial cells. Compared with the CM-(Exos-antag NC) group, lung epithelial cells cultured with CM-(Exos-miR antag) exhibited increased apoptosis, accompanied by elevated Bax and Cleaved Caspase-3 expression and reduced Bcl-2 expression (all *p* < 0.05; **Figure 5K**). Similarly, compared with the CM-(Exos + oe-NC) group, both the CM-(Exos + oe-Smad4) and CM-(Exos + oe-NLRP3) groups displayed enhanced lung epithelial cell apoptosis, increased Bax and cleaved Caspase-3 levels, and decreased Bcl-2 expression (all *p* < 0.05; **Figure 5K**).

Collectively, these findings demonstrate that depletion of Exos- derived miR-19b-3p or overexpression of Smad4/NLRP3 partially abolished the protective effects of ADMSC-Exo by restoring macrophage M1 polarization and pyroptosis, thereby promoting apoptosis of lung epithelial cells.

### 3.6 ADMSC-exo-derived miR-19b-3p inhibited NLRP3-mediated macrophage M1 polarization and pyroptosis by suppressing the TGFBR2/Smad4 axis, thereby reducing lung epithelial cell apoptosis

To further confirm whether TGFBR2 mediates the protective effects of ADMSC-Exo-derived miR-19b-3p, RAW 264.7 cells were transfected with si-TGFBR2 and subsequently co-treated with LPS and Exos-miR-19b-3p antagonist. Compared with the Exos-miR antag + si-NC group, the Exos-miR antag + si-TGFBR2 group showed reduced expression of TGFBR2, Smad4, and NLRP3 in RAW 264.7 cells (all *p* < 0.05; **Figure 6A**). Furthermore, TGFBR2 silencing decreased CD86-positive macrophages, increased CD206-positive macrophages (all *p* < 0.05; **Figure 6E**), and attenuated macrophage pyroptosis (all *p* < 0.05; **Figure 6H**).

**Figure 6:**
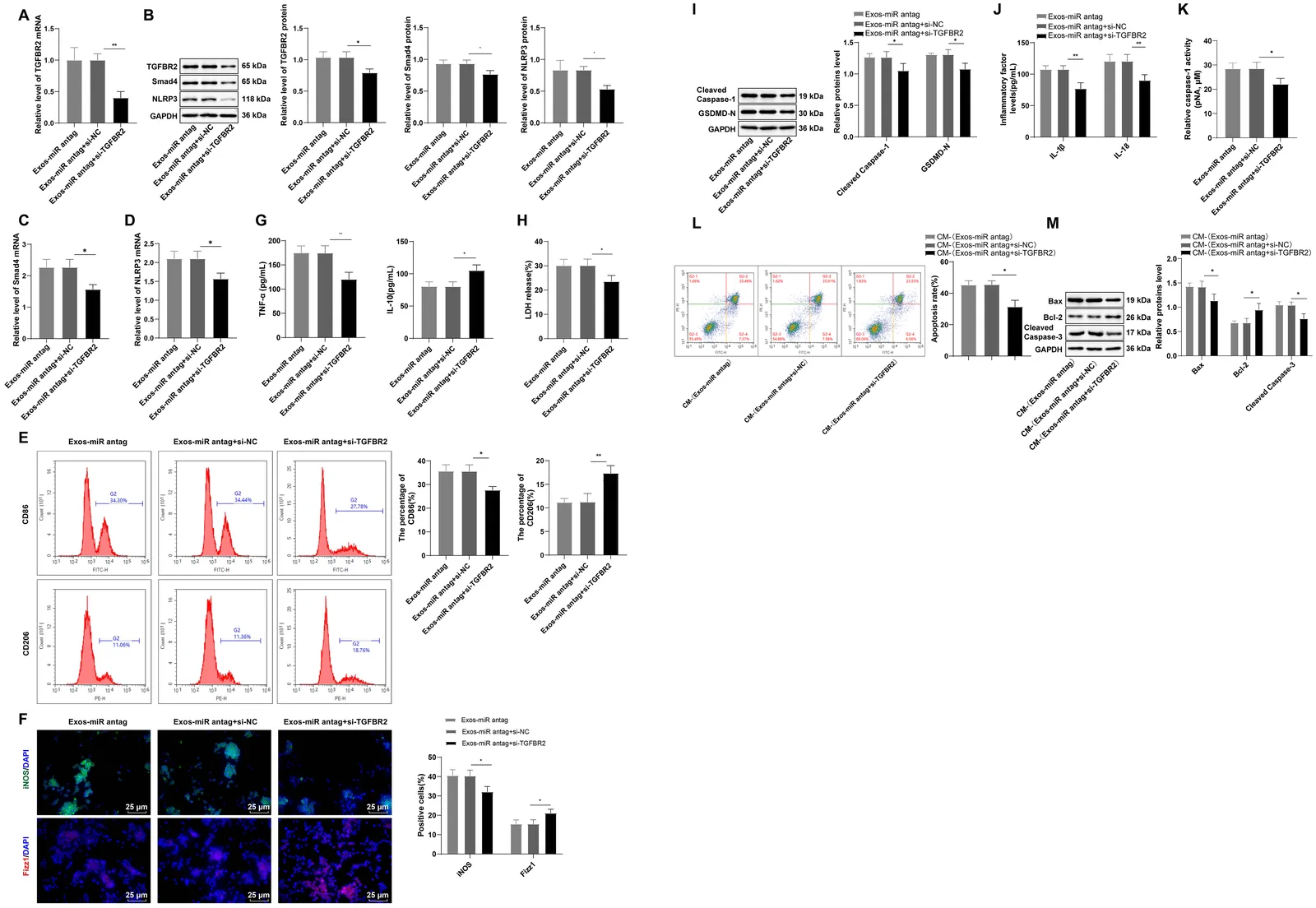
ADMSC-Exo mediated miR-19b-3p to inhibit the TGFBR2/Smad4 axis, thereby suppressing NLRP3-mediated macrophage M1 polarization and pyroptosis, ultimately alleviating lung epithelial cell apoptosis. **(A)** Determination of TGFBR2 mRNA levels using RT-qPCR; **(B)** Assessment of TGFBR2, Smad4, and NLRP3 protein expression using western blot; **(C, D)** Measurement of Smad4 and NLRP3 levels using RT-qPCR; **(E)** Evaluation of the proportions of CD86 and CD206 positive cells using flow cytometry; **(F)** Quantification of iNOS and Fizz1 in RAW 264.7 cells by immunofluorescence; **(G)** Levels of TNF-α and IL-10 in RAW 264.7 cells measured by ELISA; **(H)** LDH released from RAW 264.7 cells was detected using a kit; **(I)** Measurement of NLRP3, Cleaved Caspase-1, and GSDMD-N protein levels in RAW 264.7 cells using western blot; **(J, K)** ELISA detection of IL-1β and IL-18 levels and Caspase-1 activity in RAW 264.7 cells; **(L)** Flow cytometry assessment of lung epithelial cell apoptosis; **(M)** Western blot analyses of Bax, Bcl-2, and Cleaved Caspase-3 expression in lung epithelial cells. Cell experiments were repeated thrice. Data of the normal distribution were expressed as mean ± standard deviation. One-way ANOVA was used for multi-group data comparisons. Tukey’s multiple comparison test was implemented for *post hoc* tests. *p < 0.05, **p < 0.01. ADMSC-Exo, adipose-derived mesenchymal stem cells-exosomes; ELISA, Enzyme-Linked Immunosorbent Assay; ANOVA, analysis of Variance; NLRP3, nucleotide oligomerization domain-like receptor protein 3; Smad4, mothers against decapentaplegic homolog 4; ELISA, Enzyme-Linked Immunosorbent Assay; ANOVA, analysis of Variance; IL-1β, interleukin-1 beta; IL-18, interleukin-18; TNF-α, tumor Necrosis Factor-alpha; IL-10, interleukin-10; LDH, lactate dehydrogenase; RT-qPCR, reverse transcription-quantitative polymerase chain reaction; iNOS, inducible nitric oxide synthase.

Moreover, conditioned medium from treated macrophages was collected to culture lung epithelial cells. Compared with the CM-(Exos-miR antag + si-NC) group, cells cultured with CM-(Exos-miR antag + si-TGFBR2) exhibited reduced apoptosis, decreased Bax and Cleaved Caspase-3 expression, and increased Bcl-2 expression (all *p* < 0.05; **Figure 6L**).

Together, these results indicate that ADMSC-Exo-derived miR-19b-3p suppresses NLRP3-mediated M1 macrophage polarization and pyroptosis by targeting the TGFBR2/Smad4 axis, thereby alleviating lung epithelial cell apoptosis.

### 3.7 ADMSC-exo-derived miR-19b-3p alleviated LPS-induced ALI in mice by suppressing NLRP3-mediated macrophage M1 polarization and pyroptosis through inhibition of the TGFBR2/Smad4 axis

To validate the regulatory role of the TGFBR2/Smad4 pathway *in vivo*, LPS-induced ALI mice were treated with Exos-miR-19b-3p antagonist in combination with Ad-sh-TGFBR2. Compared with the LPS + Exo-miR antag + Ad-sh-NC group, mice in the LPS + Exo-miR antag + Ad-sh-TGFBR2 group exhibited significantly reduced expression of TGFBR2, Smad4, and NLRP3 in lung tissues (all *p* < 0.05; **Figure 7A**).

**Figure 7:**
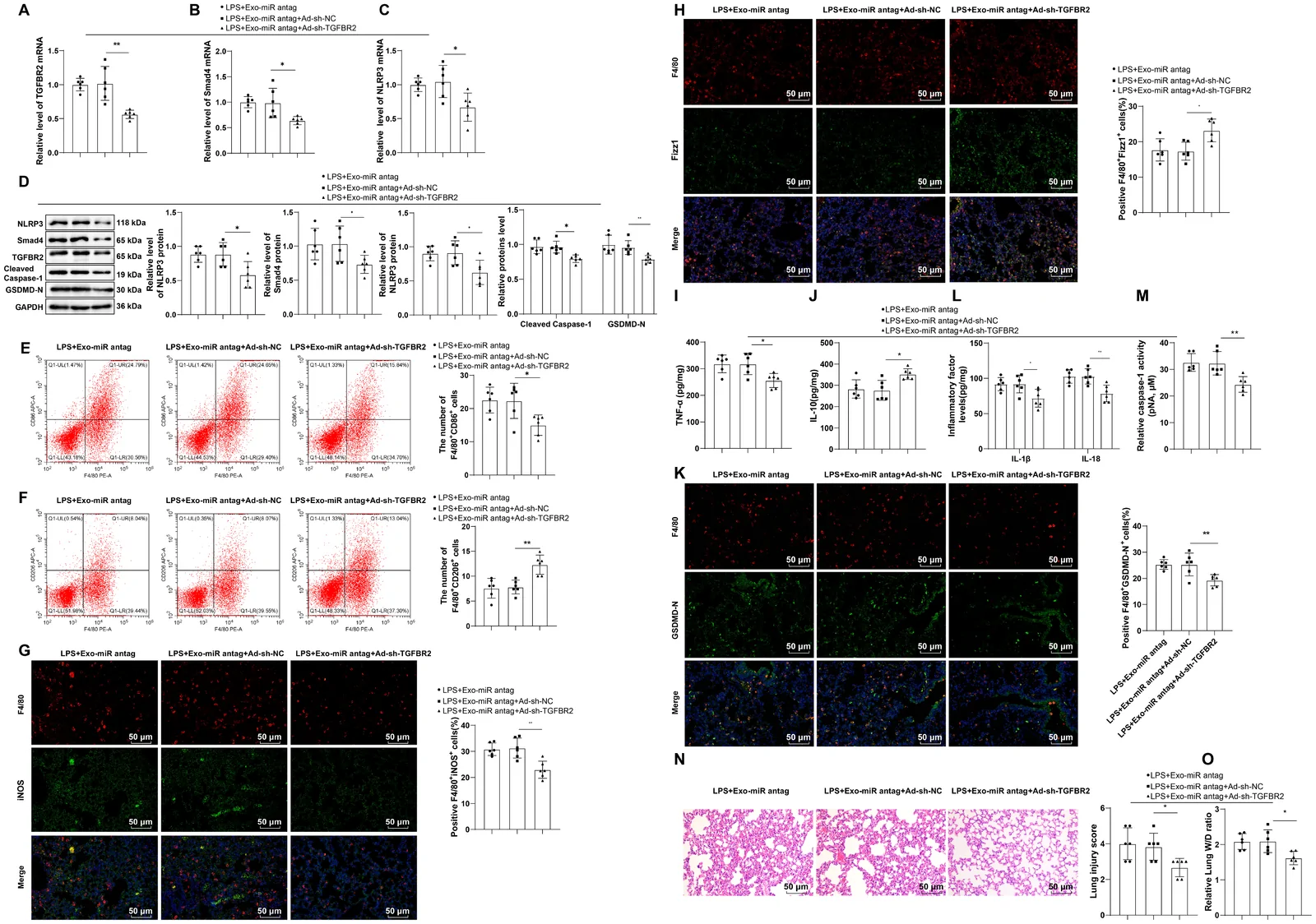
ADMSC-Exo mediated miR-19b-3p to modulate the TGFBR2/Smad4 axis, thereby suppressing NLRP3-mediated macrophage M1 polarization and pyroptosis, ultimately alleviating LPS-induced ALI in mice. **(A–C)** RT-qPCR to assess TGFBR2, Smad4, and NLRP3 levels in mouse lung tissues; **(D)** Western blot analyses of TGFBR2, Smad4, NLRP3, Cleaved Caspase-1, and GSDMD-N levels in mouse lung tissues; **(E, F)** Flow cytometry analyses of F4/80^+^CD86^+^ (M1 polarization) and F4/80^+^CD206^+^ (M2 polarization) macrophages in lung tissues; **(G, H)** Quantification of iNOS and Fizz1 in lung tissue by immunofluorescence; **(I, J)** Levels of TNF-α and IL-10 in lung tissue measured by ELISA; **(K)** Immunofluorescence staining of F4/80^+^GSDMD-N^+^ expression in macrophages in mouse lung tissues; **(L, M)** ELISA detection of IL-1β and IL-18 levels and Caspase-1 activity in mouse lung tissues; **(N)** H&E staining to detect the pathological changes of lung tissues and the lung injury score; **(O)** Pulmonary W/D ratio determination. n = 6. The data were denoted as mean ± standard deviation. One-way ANOVA was implemented for comparisons between multiple sets of data, and the Tukey’s multiple comparison test was used for *post hoc* tests. *p < 0.05, **p < 0.01. ADMSC-Exo, adipose-derived mesenchymal stem cells-exosomes; ELISA, Enzyme-Linked Immunosorbent Assay; ANOVA, analysis of Variance; NLRP3, nucleotide oligomerization domain-like receptor protein 3; Smad4, mothers against decapentaplegic homolog 4; ELISA, Enzyme-Linked Immunosorbent Assay; ANOVA, analysis of Variance; IL-1β, interleukin-1 beta; IL-18, interleukin-18; TNF-α, tumor Necrosis Factor-alpha; IL-10, interleukin-10; LDH, lactate dehydrogenase; H&E, hematoxylin and eosin; iNOS, inducible nitric oxide synthase; W/D, wet/dry.

Furthermore, TGFBR2 knockdown reversed the effects induced by Exos-miR-19b-3p inhibition, resulting in decreased macrophage M1 polarization, enhanced M2 polarization, and reduced macrophage pyroptosis (all *p* < 0.05; **Figure 7D**). In addition, TGFBR2 silencing significantly improved pulmonary pathological changes, as evidenced by reduced lung wet/dry (W/D) ratio and decreased lung injury scores (all *p* < 0.05; **Figure 7N**).

Taken together, these findings demonstrate that ADMSC-Exo delivers miR-19b-3p to suppress the TGFBR2/Smad4 signaling axis, thereby inhibiting NLRP3-mediated macrophage M1 polarization and pyroptosis and ultimately alleviating LPS-induced ALI in mice.

## 4 Discussion

Sepsis is a life-threatening pathological condition characterized by multiple organ dysfunction caused by an uncontrolled host immune response to infection, accompanied by excessive activation of inflammatory pathways and release of inflammatory mediators (42, 43). ALI is a common pulmonary complication of sepsis and contributes substantially to the high morbidity and mortality associated with sepsis-related complications (44, 45). In recent years, adipose-derived mesenchymal stem cells (ADMSCs) have emerged as a promising therapeutic strategy for ALI owing to their immunomodulatory and tissue-repair properties. ADMSC-derived exosomes (ADMSC-Exo), in particular, have been reported to alleviate LPS-induced ALI by suppressing inflammatory responses and reducing tissue injury (46, 47). Increasing evidence has highlighted the critical roles of macrophage pyroptosis and M1 polarization in the progression of ALI (48). However, the precise molecular mechanisms through which ADMSC-Exo regulate NLRP3 inflammasome-mediated macrophage M1 polarization and pyroptosis during LPS-induced ALI remain largely unclear. In the present study, we demonstrated for the first time that ADMSC-Exo alleviate LPS-induced ALI by suppressing NLRP3-mediated macrophage M1 polarization and pyroptosis through regulation of the TGFBR2/Smad4 signaling axis.

Previous studies have demonstrated that ADMSC-Exo can attenuate systemic inflammation and organ damage, thereby improving outcomes in experimental models of sepsis (49). Similarly, MSC-derived exosomes have been shown to reduce the severity of trauma-induced ALI in rats (50). Consistent with these findings, we established an LPS-induced ALI model and observed that ADMSC-Exo administration significantly reduced lung injury scores and the lung wet/dry (W/D) ratio while preserving alveolar structure integrity, indicating a protective effect against pulmonary injury (38, 47). TGFBR2 has been reported to be aberrantly upregulated in sepsis and LPS-induced inflammatory models (15). In agreement with previous observations, our study showed that TGFBR2 expression was elevated in lung tissues following LPS stimulation, whereas ADMSC-Exo effectively suppressed TGFBR2 expression. Furthermore, forced overexpression of TGFBR2 using an Ad-TGFBR2 adenoviral vector markedly weakened the protective effects of ADMSC-Exo, resulting in aggravated inflammatory infiltration, alveolar structural disruption, and increased lung injury severity. These findings suggest that TGFBR2 is an important mediator of the protective actions of ADMSC-Exo in ALI. Supporting this concept, heparin-binding protein has been reported to interact with TGFBR2 and activate TGF-β signaling, thereby promoting ALI progression (51). In addition, macrophages can release extracellular matrix-stored TGF-β2, which contributes to persistent inflammatory activation and tissue injury (52, 53). Conversely, TGFBR2 inhibition has been shown to attenuate LPS-induced sepsis and improve survival outcomes in rats (54). Collectively, these findings support the hypothesis that ADMSC-Exo exert protective effects against LPS-induced ALI, at least partially, through suppression of TGFBR2 signaling.

Macrophage polarization is a crucial determinant of inflammatory progression and tissue repair during ALI. In septic ALI, LPS stimulation induces macrophages toward a pro-inflammatory M1 phenotype and is closely associated with enhanced pyroptotic activation (40). During the early phase of ALI, M1 macrophages expressing CD86 release large amounts of inflammatory cytokines promoting neutrophil recruitment and amplifying pulmonary inflammation (55). In contrast, M2 macrophages expressing CD206 contribute to inflammatory resolution and tissue repair through secretion of anti-inflammatory mediators and growth factors, including platelet-derived growth factor and TGF-β (56). In the present study, ADMSC-Exo treatment suppressed M1 macrophage polarization while promoting M2 polarization in LPS-induced ALI mice, accompanied by reduced expression of pyroptosis-associated proteins (GSDMD-N and cleaved Caspase-1) and decreased production of inflammatory cytokines (IL-1β and IL-18). These findings are consistent with previous reports demonstrating the immunoregulatory effects of MSC-derived exosomes on macrophage phenotypic transformation and inflammatory injury (57, 58). Importantly, the beneficial effects of ADMSC-Exo were substantially reversed by TGFBR2 overexpression, indicating that TGFBR2 is functionally involved in ADMSC-Exo-mediated macrophage regulation. Furthermore, our *in vivo* and *in vitro* experiments demonstrated that TGFBR2 promotes macrophage M1 polarization and pyroptosis. Increased TGFBR2 expression in macrophages following ADMSC-Exo treatment enhanced lung epithelial cell apoptosis, as evidenced by increased Bax and cleaved Caspase-3 expression and reduced Bcl-2 levels. These findings are consistent with previous studies implicating TGFBR2 in apoptotic regulation (59). Taken together, our data indicate that ADMSC-Exo protect against LPS-induced ALI by inhibiting TGFBR2-mediated macrophage M1 polarization and pyroptosis, thereby reducing secondary lung epithelial cell apoptosis.

Previous studies have demonstrated that TGFβR2 is regulated by multiple miRNAs, including miR-20a-5p and miR-425 (60, 61). Increasing evidence indicates that miR-19b-3p expression is reduced in patients with sepsis and that restoration of miR-19b-3p expression alleviates LPS-induced inflammatory injury (62). Moreover, miR-19b-3p has been shown to regulate macrophage polarization through targeting of PKNOX1, thereby protecting against Coxsackievirus B3-induced inflammation and myocardial injury. In the present study, bioinformatics analysis and experimental validation identified TGFBR2 as a direct target of miR-19b-3p. Importantly, depletion of miR-19b-3p in ADMSC-Exo resulted in increased TGFBR2 expression, which promoted macrophage M1 polarization and pyroptosis in LPS-induced ALI mice, partially reversing the protective effects of ADMSC-Exo. Consistent with this regulatory pattern, miR-155-5p derived from human ADSC-Exos has been reported to enhance autophagy and inhibit pyroptosis in nucleus pulposus cells by targeting TGFBR2, thereby alleviating intervertebral disc degeneration (63). Collectively, these findings suggest that ADMSC-Exo exerts anti-inflammatory and tissue-protective effects through delivery of functional miRNAs, and in the context of ALI, miR-19b-3p-mediated suppression of TGFBR2 represents a critical mechanism underlying the regulation of macrophage inflammatory responses.

Upon ligand binding, TGFBR2 activates TGF-β signaling by phosphorylating receptor-associated Smad2/3, which subsequently forms a transcriptional complex with the common mediator Smad4 to regulate downstream gene expression (64). In this study, we demonstrated that TGFBR2 positively regulated Smad4 expression, consistent with previous findings (65), whereas ADMSC-Exo inhibited this signaling cascade through miR-19b-3p delivery. Furthermore, luciferase reporter and ChIP assays confirmed that Smad4 directly binds to the promoter region of NLRP3 and enhances its transcription, revealing a previously unrecognized regulatory link between the TGFBR2/Smad4 pathway and NLRP3 inflammasome activation in macrophages. The NLRP3 inflammasome is a key innate immune signaling platform that interacts with the adaptor protein ASC to activate caspase-1, subsequently promoting GSDMD cleavage and pyroptotic cell death in macrophages (66). In addition to its role in pyroptosis, NLRP3 activation has been implicated in promoting M1 macrophage polarization and inflammatory amplification in tissue injury models (67). Based on these observations, we proposed that the TGFBR2/Smad4 axis may regulate NLRP3-dependent macrophage polarization and pyroptosis, thereby influencing secondary epithelial cell injury during ALI.

Our rescue experiments further supported this hypothesis. Overexpression of Smad4 or NLRP3 partially abolished the inhibitory effects of ADMSC-Exo on macrophage M1 polarization and pyroptosis, while silencing TGFBR2 reversed the effects caused by miR-19b-3p inhibition. Moreover, suppression of TGFBR2 reduced Smad4 and NLRP3 expression, accompanied by decreased CD86^+^ M1 macrophages, increased CD206^+^ M2 macrophages, and attenuated pyroptotic activation. These findings confirm the sequential regulatory relationship within the miR-19b-3p/TGFBR2/Smad4/NLRP3 signaling cascade. Therefore, our study provides evidence that ADMSC-Exo deliver miR-19b-3p to inhibit TGFBR2/Smad4-mediated transcriptional activation of NLRP3, thereby suppressing macrophage M1 polarization and pyroptosis and ultimately reducing alveolar epithelial cell apoptosis during LPS-induced ALI.

In conclusion, this study demonstrates that ADMSC-Exo alleviates LPS-induced ALI by delivering miR-19b-3p to suppress the TGFBR2/Smad4 signaling axis, resulting in reduced NLRP3-mediated macrophage M1 polarization and pyroptosis. These findings highlight ADMSC-Exo and their cargo miRNAs as potential therapeutic candidates for sepsis-associated lung injury.

## 5 Limitations

Several limitations of this study should be acknowledged. First, although our findings identify a novel ADMSC-Exo-mediated regulatory pathway in ALI, the current study was primarily conducted using murine models. Further validation using clinical samples from patients with sepsis-associated ALI is required to determine the translational relevance of the miR-19b-3p/TGFBR2/Smad4/NLRP3 axis in humans.

Second, this study mainly focused on the molecular mechanism by which ADMSC-Exo regulates macrophage inflammatory responses through the TGFBR2/Smad4 axis. Consequently, the evaluation of systemic inflammatory responses and vascular permeability was limited, and only pulmonary edema was assessed using the lung wet/dry ratio. Future studies incorporating comprehensive assessments of vascular leakage, circulating inflammatory mediators, and extrapulmonary organ effects will provide a more complete understanding of the therapeutic potential of ADMSC-Exo in sepsis.

Third, due to limitations associated with sample collection, bronchoalveolar lavage fluid (BALF) was not obtained in the current study, preventing direct analysis of alveolar immune cell composition and cytokine profiles. Future investigations should evaluate BALF-derived inflammatory mediators and immune cell populations to further clarify how ADMSC-Exo reshape the pulmonary immune microenvironment during ALI.

Fourth, there existed a technical limitation in the flow cytometry staining panel for macrophage polarization detection. In our flow cytometric assay, both M1 (CD86^+^) and M2 (CD206^+^) macrophages were assessed using the same fluorophore (APC), without simultaneous evaluation of CD86 and CD206 expression. This strategy may have resulted in an overestimation of macrophage polarization. Future studies should employ distinct fluorophores for CD86 and CD206 to enable their simultaneous detection, thereby improving the discrimination of M1 and M2 macrophage populations and reducing potential double-positive artifacts.

## Data Availability

All data generated or analysed during this study are included in this article. Further enquiries can be directed to the corresponding author.
