# Supplementary material for: Adipose-derived mesenchymal stem cell-exosomes attenuate lipopolysaccharide-induced acute lung injury in mice by regulating the TGFBR2/Smad4 axis to suppress NLRP3-mediated macrophage M1 polarization and pyroptosis
Source: Front Immunol. 2026 Sep 14;17:1739115. doi: 10.3389/fimmu.2026.1739115 (PMC13616780; doi:10.3389/fimmu.2026.1739115)
Supplement: Supplementary file 1 [file DataSheet3.pdf]

frontiers

The International Society for the Advancement of Cytometry (ISAC) has highlighted the importance of including comprehensive methodological information to ensure data reproducibility and reliability. In line with this, Frontiers in Immunology now requires authors to submit a checklist for manuscripts that involve flow or mass cytometry. This checklist helps standardize the reporting process, improving the quality and transparency of published data. By doing so, we support scientific progress, making it easier for other researchers to replicate and validate experiments.

This form should be submitted with any manuscripts using flow or mass cytometry.

Sample/specimen/material description

- [ ] Total blood
- [ ] PBMCs
- [ ] Organ digests

Other: mouse lung tissue and RAW 264.7 cells

Did the samples suffer any treatment before or after incubation with the antibodies?

- [ ] Drug
- [x] Cell permeabilization: Triton X-100
- [x] Dye: Fixable Viability Stain (FVS)、Annexin V-FITC
- [x] Propidium iodine
- [ ] Not applicable

Other

Instrument and antibodies

Name of the Cytometer: FACSCalibur flow cytometer (Becton Dickinson, USA)

Antibodies and targets	Fluorochrome/ Metal	Catalog number/Company
e.g. anti-CD4	FITC	Cat. XXX/ XXX ltd.
anti-CD86	FITC	Cat.ab234237/Abcam

anti-F4/80	PE	Cat.ab237335/Abcam
anti-CD206	PE	Cat.98031-1-RR/Protein tech
anti-CD86	APC	Cat.ab326464/Abcam
anti-CD206	APC	Cat.G4068/Selleck
anti-CD29	FITC	Cat.G0571/Selleck
anti-CD34	FITC	Cat.G0121/Selleck
anti-CD44	FITC	Cat.ab25064/Abcam
anti-CD54	FITC	Cat.ab210225/Abcam

Data analyses

1. Name of the software FlowJo software (version X 0.7)、SPSS 21.0 (SPSS, Inc., Chicago, IL, USA)
2. Reference gating strategy in the manuscript or supplementary material

Gating strategy in (eg Figure X) For specific details, please refer to the methods section.
