# Supplementary material for: Adipose-derived mesenchymal stem cell-exosomes attenuate lipopolysaccharide-induced acute lung injury in mice by regulating the TGFBR2/Smad4 axis to suppress NLRP3-mediated macrophage M1 polarization and pyroptosis
Source: Front Immunol. 2026 Sep 14;17:1739115. doi: 10.3389/fimmu.2026.1739115 (PMC13616780; doi:10.3389/fimmu.2026.1739115)
Supplement: Supplementary file 2 [file Table2.docx]

Supplementary file 1. A total of 1455 miRNAs was identified in mouse-derived Exos in ExoCatrta database.

Supplementary file 2. Upstream miRNAs targeting TGFBR2 were predicted using the StarBase.

Supplementary file 3. Upstream miRNAs targeting TGFBR2 were predicted using the miRDB.

Supplementary file 4. Upstream miRNAs targeting TGFBR2 were predicted using the miRWalk.

Supplementary file 5. Seven differentially expressed miRNAs associated with sepsis were retrieved published researches.

Supplementary file 6. miR-19b-3p was obtained from the intersection of the jvenn online website.
