# Supplementary material for: Adipose-derived mesenchymal stem cell-exosomes attenuate lipopolysaccharide-induced acute lung injury in mice by regulating the TGFBR2/Smad4 axis to suppress NLRP3-mediated macrophage M1 polarization and pyroptosis
Source: Front Immunol. 2026 Sep 14;17:1739115. doi: 10.3389/fimmu.2026.1739115 (PMC13616780; doi:10.3389/fimmu.2026.1739115)
Supplement: Supplementary file 3 [file Table1.doc]

**Table 1** Primer sequences of each gene

| Gene | Forward 5’-3’ | Reverse 5’-3’ |
| --- | --- | --- |
| TGFBR2 | GTAGCTCTGATGAGTGCAATGAC | CAGATATGGCAACTCCCAGTG |
| miR-19b-3p | TGTGCAAATCCATGCAAAAC | CTCAACTGGTGTCGTGGAGTC |
| Smad4 | ACTGTGGATGGCTATGTGGAT | CCTTTGCCTATGTGCAACCT |
| NLRP3 | CACCTCTTCTCTGCCTACCTG | AGCTGTAAAATCTCTCGCAGT |
| GAPDH | CCACCTTCATCTTACCATTTATTATCGC | TTTGATGTTAGTGGGGTCTCG |
| U6 | ATTGGAACGATACAGAGAAGATT | GGAACGCTTCACGAATTTC |
